# Body size measuring techniques enabling stress-free growth monitoring of extreme preterm infants inside incubators: A systematic review

**DOI:** 10.1371/journal.pone.0267285

**Published:** 2022-04-22

**Authors:** Ronald H. J. van Gils, Linda S. G. L. Wauben, Onno K. Helder

**Affiliations:** 1 Division of Neonatology, Department of Pediatrics, Erasmus MC University Medical Centre, Rotterdam, The Netherlands; 2 Department of Create4Care, Erasmus MC University Medical Centre, Rotterdam, The Netherlands; 3 Research Centre Innovations in Care, Rotterdam University of Applied Sciences, Rotterdam, The Netherlands; 4 Institute of Engineering & Applied Science, Rotterdam University of Applied Sciences, Rotterdam, The Netherlands; 5 Department of Biomechanical Engineering, Faculty of Mechanical, Maritime and Materials Engineering, Delft University of Technology, Delft, The Netherlands; Jadavpur University, INDIA

## Abstract

**Introduction:**

Growth monitoring of preterm infants is essential for assessing the nutritional effects on their growth. The current growth monitoring techniques are too stressful, however, for the smallest preterm infants. We performed a systematic review to summarize studies on stress-free techniques for measuring the body size of preterm infants inside incubators other than the traditional calliper and tape measure-based instruments.

**Methods:**

We searched four online literature databases: Embase, Medline, Web of Science Core Collection, and Cochrane, using search terms related to patients (neonates, infants, children) and body size measuring techniques. By means of expert judgement we assessed the techniques’ suitability for stress-free body size measurement of an infant lying in an incubator. As a criterion for suitability, we used an imaginary ideal technique.

**Results:**

Twenty-six studies were included in this review. In 24 studies, the technique for body size measurement was related to 3D technology, and the majority of these studies acknowledged clinical superiority of 3D over 2D data. Two 3D techniques were assessed as suitable for stress-free measurement of preterm infants inside incubators. The first technique used a commercially available 3D handheld scanner which needed 3D postprocessing to derive measurement data. The second technique used a self-developed stereoscopic vision system.

**Conclusions:**

3D volumetric parameters have higher clinical value for growth monitoring than 2D. In addition, contactless 3D measurements enable stress-free growth monitoring of even the smallest preterm infants. However, the time-consuming 3D postprocessing challenges the usability of 3D techniques. Regrettably, none of the identified suitable 3D techniques met all our requirements of an ideal all-in-one body size measuring technique for extreme preterm infants. Handheld 3D scanning might have the best properties for developing this ideal technique.

## Introduction

**Fig 1:**
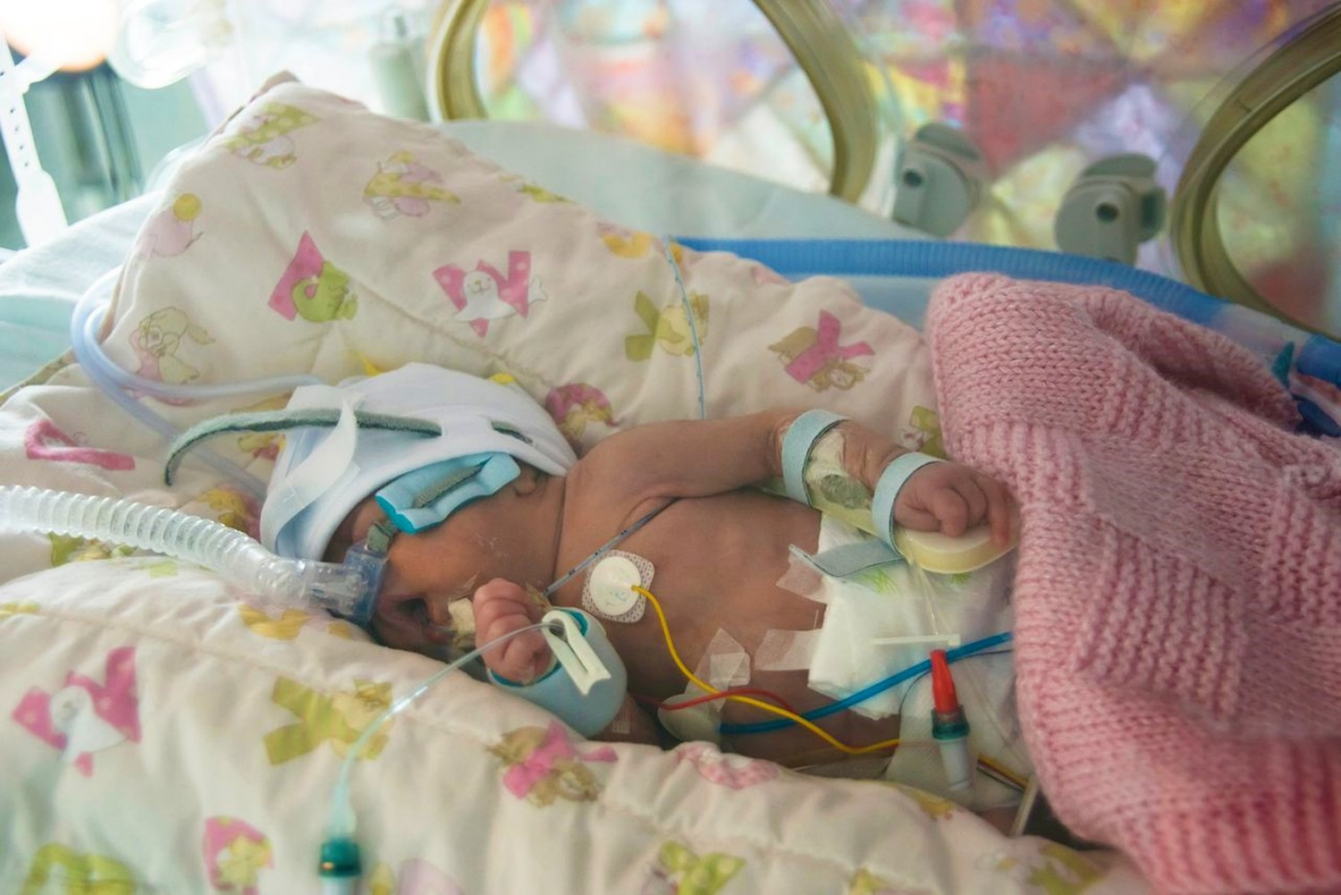
Preterm infant lying inside an incubator at a neonatal intensive care unit (NICU). The limited space inside the incubator and the “spaghetti of wires, lines and tubes” makes it difficult to measure the body size without causing stress to the infant. (image: CC BY BMJ Bonner et al. 2016).

**Fig 2:**
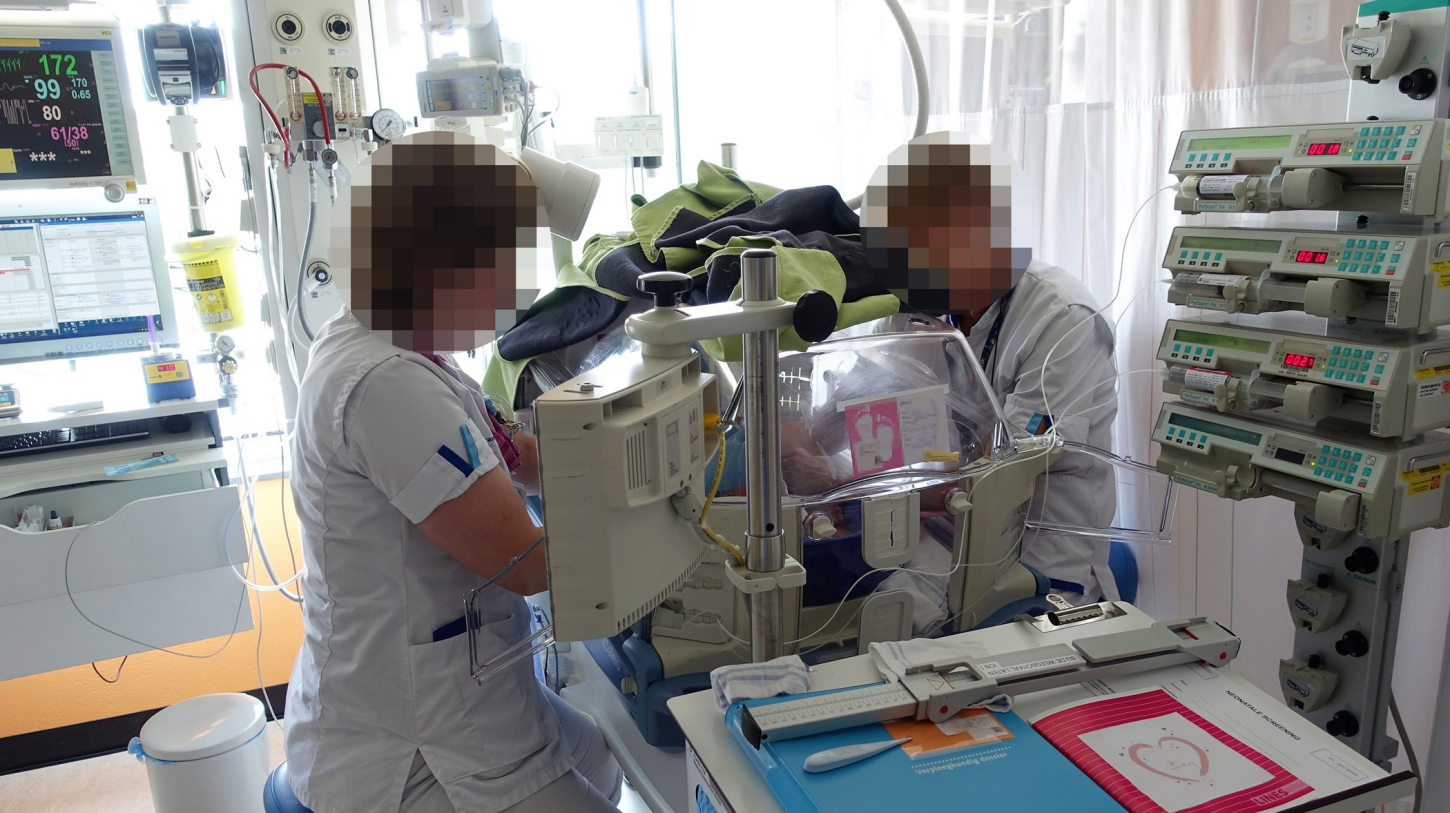
Growth reference charts are based on two parameters: Body length (BL) and head circumference (HC). As both BL and HC are expressed in centimetres with one decimal, measuring instruments with an accuracy of 1 mm are clinically sufficient.

Preterm infants–with birthweight less than 1500 grams–require optimal growth to foster the short- and long-term neuro-developmental outcomes [1–3] (Fig 1). They may have difficulty, however, to effectively convert nutrition into energy for actual growth, for example in the case of infectious disease. A study has found that personalized nutrition may prevent a preterm infant’s growth failure [4]. To assess the effect of personalized nutrition on growth, the infant’s body size and weight should be accurately and frequently measured, ideally starting directly after birth. Growth reference charts of weight, body length and head circumference are currently seen as the golden standard to assess growth (Fig 2) [5, 6].

Stress, induced by pain or discomfort, is related to suboptimal brain development [7, 8] and negative effects for health in later life [9–13]. Neonatal stress is mainly related to procedural pain from interventions in the neonatal intensive care unit (NICU), such as skin breaking procedures. Furthermore, any form of discomfort caused by light [14], noise [15–18] or touching may induce stress and can disturb the wake-sleep cycle [19, 20].

Achieving absence of stress and discomfort is essential in the routine care of preterm infants. Today, individualized care programs with a strong focus on prevention of stress are accepted as standard NICU care [21–23]. Routine caregiving is matched with the infant’s sleep-wake cycle, and performed as minimally disturbing as possible. Infants should remain in their comfortable, supportive ‘snuggles’ as much as possible, with their legs curled up, which is their most comfortable, natural position.

**Fig 3:**
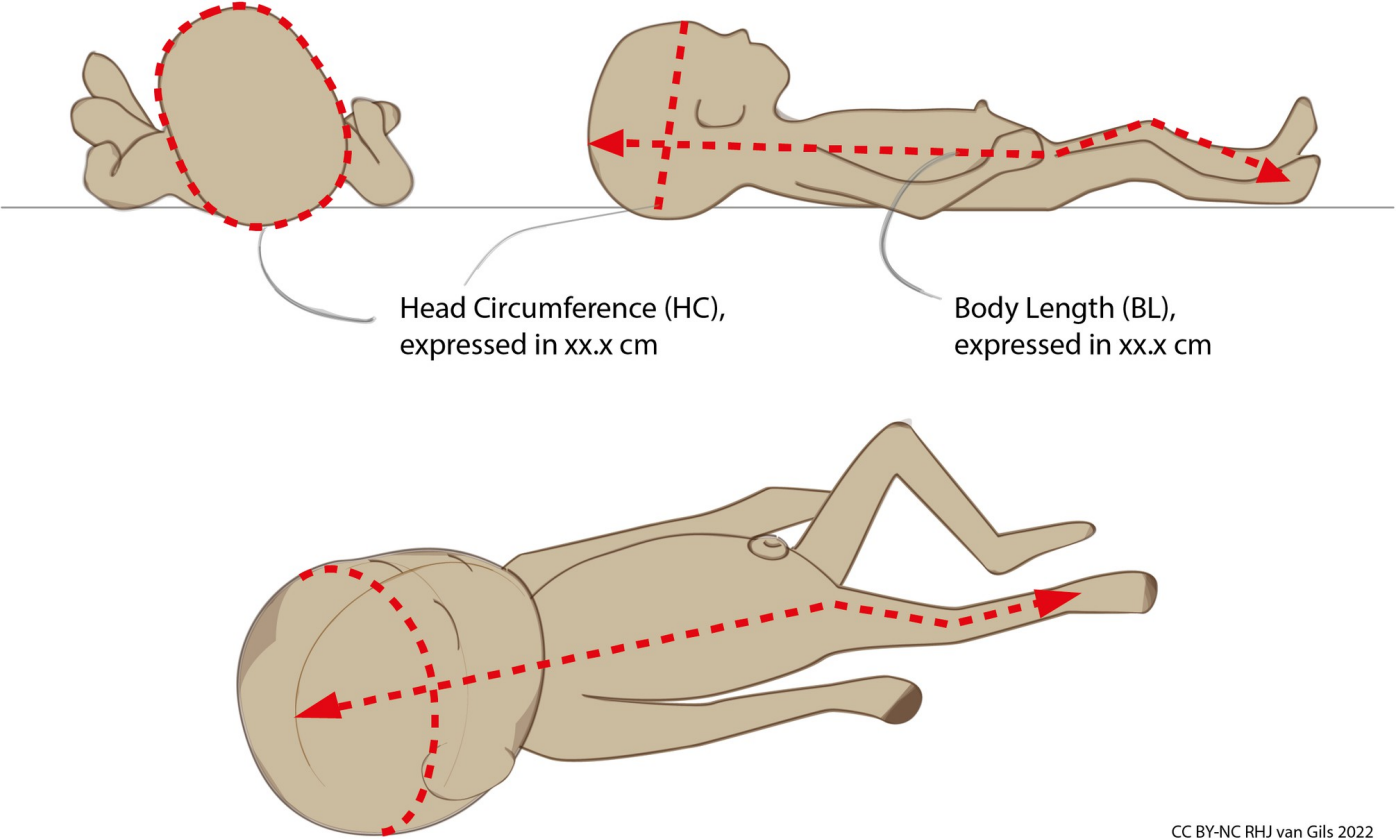
An incubator bedspace at a neonatal intensive care unit (NICU). Body size measurements for growth monitoring are mostly performed by two NICU nurses in tandem. They work from opposite sides, and through small openings in the transparent cover to prevent a temperature drop.

Measuring body length or head circumference with commonly used calliper-style or tape measure-style instruments techniques can be so stressful, however, for very small or sick preterm infants that NICU may tend to simply skip measurements [24–27]. These instruments must be brought inside the tiny space of the incubator, during which undesired contact with the infant, the tubes and the lines is nearly unavoidable (Figs 1 and 3). Repositioning of these ventilation and gastric feeding tubes in the nose, monitor wires taped to skin and venous catheters in hand, feet or belly can be painful or uncomfortable. Furthermore, stretching the infants’ curled-up legs, which is necessary to measure body length with calliper or tape measure, is uncomfortable for them [24].

**Fig 4:**
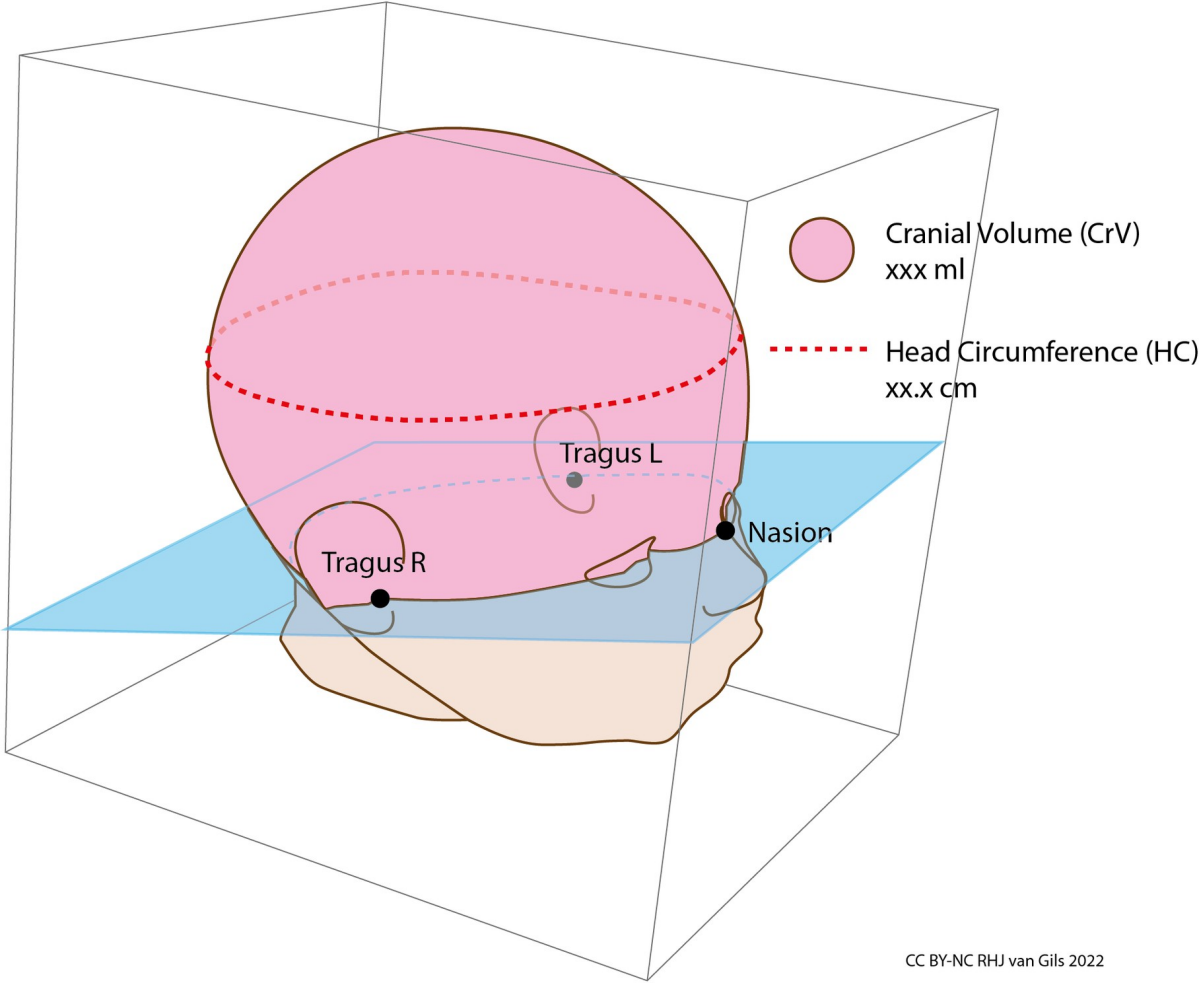
3D Cranial volume (CrV) measurement could provide more clinically relevant growth data than 2D HC measurement. CrV is mostly defined by the volume above a virtual plane through three anatomical points: tragus left, tragus right and nasion. CrV growth reference charts are being developed, with CrV expressed in millilitres (ml) in a round number. CrV measuring instruments with an accuracy of 1 ml should be clinically sufficient.

The paradox that accurate growth monitoring is essential, but too stressful for the most vulnerable preterm infants with the currently used calliper-style or tape measure-style instruments, led to our search for stress-free techniques for growth monitoring. The use of contactless 2D or 3D technology seems logical to minimize disturbance of the infant. 3D body size parameters, such as cranial volume, could provide more accurate growth data than current 2D-data (Fig 4). An overview and comparison of studies related to such techniques was missing in the literature. Therefore, we performed a systematic review to explore suitable techniques for measuring 2D and 3D body size parameters of preterm infants in their incubators, without causing stress to the infant.

## Materials and methods

### Design

**Fig 5:**
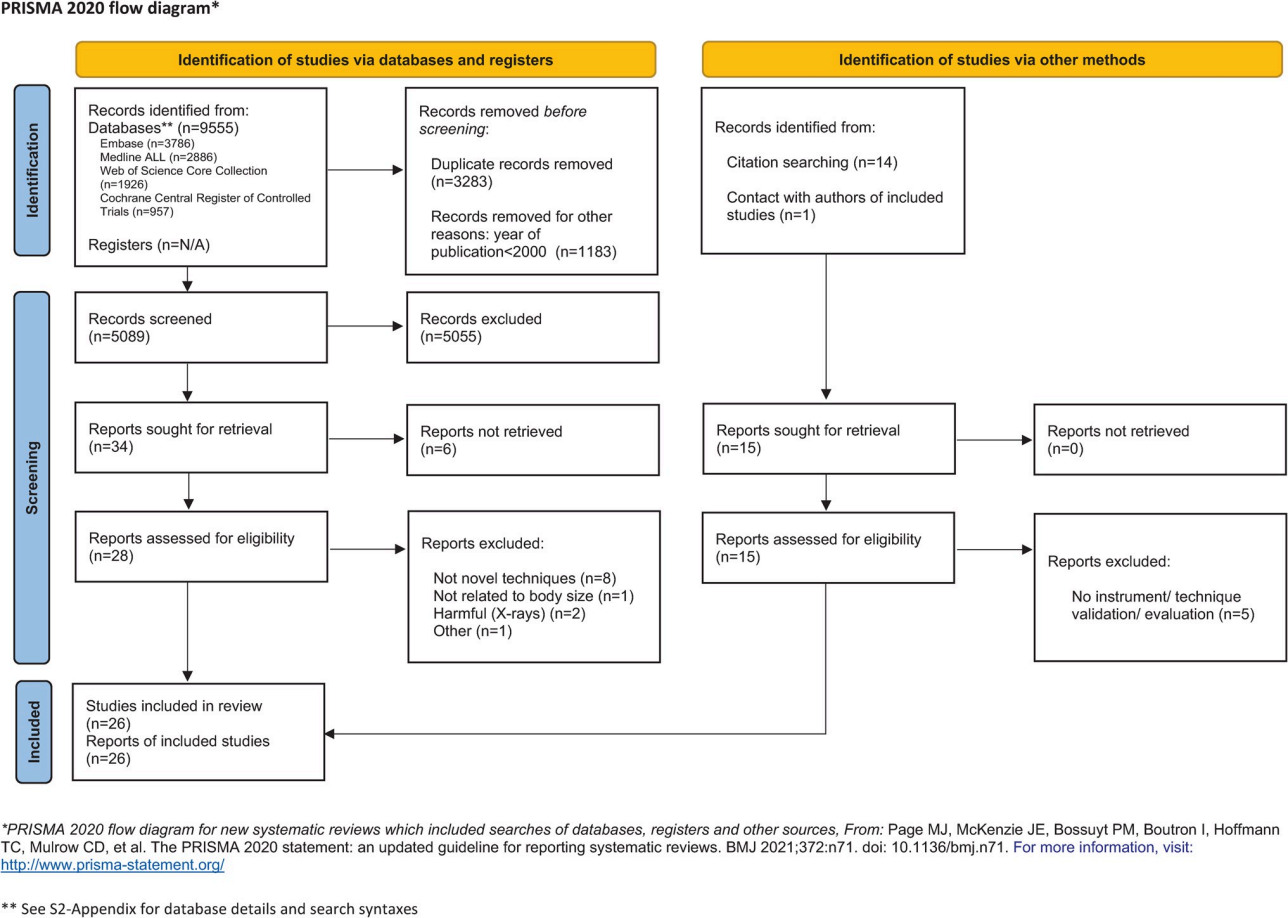
PRISMA 2020 flow diagram for systematic reviews.

The search strategy was designed to identify studies with techniques for measuring body size parameters of neonates. Our data collection method was designed for qualitative assessment of techniques’ suitability for stress-free growth monitoring of preterm infants lying in incubators. As a consequence of our aims and methods, statistical meta-analysis of data was not applicable. The Preferred Reporting Items for Systematic Review and Meta-Analysis Protocols (PRISMA-P) served as design guideline [28]. The PRISMA flow diagram is presented in Fig 5 and the PRISMA checklist as appendix S1 Checklist.

### Eligibility criteria

Our eligibility criteria filtered out studies that use, describe, or evaluate techniques for measuring body size parameters. These techniques should be, or could be, potentially suitable for stress-free growth monitoring of infants lying in incubators. Therefore, the following eligibility criteria, related to technique characteristics, steered the inclusion of articles:

- Techniques should measure any kind of body size parameter, such as body length and head circumference, including volumetric parameters such as cranial volume.
- Techniques should be potentially stress-free. We excluded body size measuring techniques that rely on patient-instrument contact, mostly tape measure or calliper-style instruments [25, 29].
- Techniques should enable bedside measuring. We excluded non-portable, room-bound techniques, such as MRI, as not suitable for (incubator) bedside monitoring. However, we included stationary techniques if we agreed that it can be potentially made mobile to fit at an incubator bedspace.
- Techniques should not be invasive or harmful. For example, X-ray technology was excluded.
- Techniques should be capable to measure small infants. The age range was extended beyond neonatal age, including preterm infants, and older children (< 18 years), because novel measuring techniques used in anthropometric studies with older children might be, or might be made suitable for preterm infants. Studies evaluating techniques that met all criteria, but used reference objects such as mannequin heads or plaster models instead of patients, were included.

In addition, we added eligibility criteria for study design and report characteristics. We included all types of study designs. Included were journal articles and seminar proceedings, written in English, Dutch or German. Because we aimed to identify novel techniques, we limited the year of publication to 2000 until 16 July 2021.

### Search strategy

An electronic search was performed, with last update on 16 July 2021, in four online literature databases: Embase, Medline, Web of Science Core Collection, Cochrane. The search syntax for the electronic search was constructed on keywords related to patients (neonates, infants, children), interventions (body size measurement), comparators (techniques), outcomes (validity) and known articles that fully matched our eligibility criteria. Details of the databases, including the search syntaxes and the number of records identified from each database, can be found in S1 Appendix.

### Selection and quality assessment

Three reviewers (RG, LW, OH) selected eligible articles in three steps. All selection steps were independently performed by two reviewers, dividing records over two pairs of reviewers (RG+LW and RG+OH). When a pair of reviewers disagreed about inclusion, consensus was reached after discussing the article’s eligibility with a third reviewer. In the first step, the titles and abstracts of the records identified in the electronic search were screened for potential eligibility. In the second step, the full texts of the potentially eligible articles were assessed on merit. Articles that did not meet the eligibility criteria were discarded. The remaining articles were included in this review. In the third step, for all included articles, cross references and references that cited the article (‘cited-by’) were screened to identify possible extra eligible records. These cross and cited-by references were first screened on title and abstract for eligibility. The potentially eligible articles from this screening were then assessed full text. The resulting eligible cross and cited-by references were added to our list of included articles. The methodological quality of all included articles was assessed using the ‘QualSys’ tool for Standard Quality Assessment Criteria for Evaluating Primary Research Papers from a Variety of Fields [30].

### Data collection

A data extraction form was constructed to facilitate assessment of articles and to structure data collection. Data fields of this data extraction form were related to the inclusion criteria. Data of the included articles were independently collected by a pair of reviewers. Collected data of both reviewers were merged in one final data extraction form. Data is reported in narrative form. Filling out some of these data fields required the reviewers’ expert judgement; for example, the suitability of a technique for use in a NICU-setting.

### Assessment of techniques’ suitability

Expert judgement, based on interpretation of collected data, was used to assess the techniques’ suitability for measuring the body size of preterm infants lying in an incubator. This suitability was assessed by expert reviewers (RG, LW, OH). To guide this assessment, we envisioned a technique with ideal properties.

This ideal technique employs an all-in-one device that can measure body length, head circumference, and cranial volume of ventilated preterm infants lying in incubators. The technique should include volumetric measurements of cranial volume thereby providing more clinically relevant growth data than the head circumference. Accuracy and reliability of measurements should be sufficient for clinical use. Based on growth reference charts, this accuracy should be at least 1 mm for body length and head circumference [31] and 1 ml for cranial volume [32]. Moreover, to be truly ideal, the accuracy and reliability of this device should not be user-dependent. The device is mobile and compact to fit at the NICU incubator-bedspace. The device measures through the transparent incubator cover, without the necessity to open doors or remove the cover. To be as non-disturbing as possible, no extra preparation or repositioning of the infant during measurements is needed other than the routine care handling.

These ideal properties formed five requirements to rate techniques with discrete scores, presented in Table 1. Minimum scores per requirement were defined to rate techniques in three classes: ideal, suitable, potentially suitable. Minimum scores must be met independently: a criterium’s high score cannot compensate a another criterium’s low score. Techniques not fitting in one of the three classes were rated as not suitable. A narrative motivation clarified the techniques’ classification.

**Table 1:** Properties to assess the techniques’ suitability for measuring the body size of a preterm infant lying in an incubator.

Technique property	How to rate included studies	Scores for ideal device	Minimum score to rate as suitable	Minimum score to rate as potentially suitable
Measures ventilated or respiratory supported preterm infants lying in an incubator	0 = not reported or not feasible^a^	3	1	1
	1 = not reported but reasonable belief^b^ in feasibility			
	2 = reported measurements of infants in incubator			
	3 = reported measurements of ventilated infants in incubator			
Sufficient accuracy^c^ for clinical use, and accuracy not influenced by user-actions	0 = no data available in study or reported as not sufficient	3	2	1
	1 = as 0, but reasonable belief^b^ in feasibility			
	2 = reported or assessed as sufficient			
	3 = sufficient and not influenced by user-actions			
Measures through incubator’s transparent cover, without opening doors or removing cover	0 = not reported or not feasible^a^	3	2	1
	1 = not reported but reasonable belief^b^ in feasibility through open doors or closed cover			
	2 = yes, but through open doors			
	3 = yes, through closed cover			
No extra preparation or repositioning of the infant needed for measurement, other than routine care handling	0 = yes or not reported	3	2	1
	1 = yes, but reasonable belief^b^ that extra handling could be combined with routine care			
	2 = yes, but extra handling was combined with routine care			
	3 = no preparation or reposition needed at all			
Can measure CrV, besides BL and HC, and ideally with one device	0 = no reported measurements of BL, HC, or CrV	3	1	1
	1 = BL and/or HC			
	2 = CrV			
	3 = CrV, HC, and BL with one device			

^a^ This also includes all stationary devices that are too large or immobile to use at an incubator: these techniques are rated as not suitable.

^b^ Reasonable belief can be based on additional information obtained via contact with authors of included studies, or the experts’ judgement.

^c^ Sufficient accuracy for body length (BL) and head circumference (HC) is 1 mm, and for cranial volume (CrV) 1 ml.

### Contact with authors

Collected data from an article were communicated to the corresponding author for verification. Author’s comment was asked for each data field. If the author had not responded after 14 days, a reminder was sent. The author’s comments were added to our data extraction forms with the pre-fix: ‘Author’s comment:’. This pre-fix was omitted if the comments concerned minor administrative changes (e.g., the department involved).

### Presentation of qualitative analyses of included studies

All collected data and data analyses were tabulated in an All-data-table (S1 Data). To structure the data presentation, four Data-tables were constructed from this All-data-table, each covering a specific topic. We have inserted summarizing tables in the main text to facilitate a quick overview of quantitative information about studies.

## Results

### Search and selection outcomes

Fig 5 visualizes results of the search and selection process in a PRISMA flow diagram. The electronic search yielded 5189 records to be screened. Screening of titles and abstracts led to 34 articles for full text assessment. Fifteen additional records from other sources were added to our full text assessment: fourteen identified by screening of cross and cited references of eligible articles and one through contact with authors. Finally, twenty-six studies matched the eligibility criteria and were included in this review [32–57]. Comments were received from the corresponding authors of 18 articles. These comments were added to the data extraction forms.

### Characteristics of studies & methodological quality

S2 Data presents the study-characteristics and QualSys scores for methodological quality per study. Full details can be found in the S1 Data. Twenty-four studies were performed at university medical centres or medical schools; two at technical universities [40, 42]. Across all studies, the ages of the patients involved ranged from 24 weeks gestational age to 12 years of age. Only four studies concerned preterm infants [33, 43, 50, 53]. In general, QualSys scores are high, reflecting good methodological quality, and the individual scores of the two reviewers correlate. Both reviewers rated the methodological quality of one study [40] very poor (0.06 and 0.07), as the technology readiness level of the prototype evaluated in this study was low and the documentation was poor.

### Aims of studies

Table 2 categorizes the aims of the studies, related to technology and clinical aspects and body size parameters. Some studies had more than one aim. Almost half of all studies compared 3D scanning with traditional manual anthropometric measuring techniques, such as calliper, tape measure or length board.

**Table 2:** Categorized aims of studies.

Technology-clinical	Number of studies	Type of body size parameter	Number of studies
3D scanning versus manual	12	Head: Head circumference	13
3D scanning versus 3D reference scan of plaster impression model	2	Head: Head volume, Cranial volume, Intracranial volume	13
3D scanning versus X-ray	2	Head: Head shape/ dimensions	10
3D scanning, Head Shape Analysis: Growth analysis	3	Head: Face shape/ dimensions	4
3D scanning, Head Shape Analysis: Treatment planning	2	Body length (crown-heel length, total body length, stature, height)	4
3D scanning, Head Shape Analysis: Operative results	1	Upper Arm Circumference	1
Correlation Head circumference and Cranial volume	3	Body surface area	1
Ultrasonic (prototype evaluation)	1		
2D linear metric via photographs	1		
Anthropometric data collection	1		

Two studies measured multiple body size parameters: one both body length and head circumference [33], and one ‘standard anthropometric data’ (body length, head circumference, upper arm circumference) [38]. One study measured the total body surface area [52].

### Type of technology

S3 Data presents the used technologies in studies with specifications and data as recorded by the authors. Type of 3D scanning-technique and -devices were structured using four characteristics [58], described and visualized in S2 Appendix. Table 3 presents an overview of used 3D scanners.

**Table 3:** Used 3D scanner device models.

3D scanner manufacturer (device model)	Handheld or stationary/desktop	Number of studies
3dMD Cranial System	Stationary	5
3dMD Face System	Stationary	2
Orthomerica STARscanner	Stationary, desktop	3
3D-Shape	Stationary	1
3D-Shape, custom-built	Stationary	1
Fuel3D Scanify	Handheld	2
Smartphone 3D scanning (via slow-motion video)	Handheld	2
VECTRA H1	Handheld	1
OMEGA^a^	Handheld	1
M4D^a^	Handheld	1
Structure Sensor with iPad	Handheld	1
	Total 3D scanners in all studies	20
	Total Stationary in all studies	12
	Total Handheld in all studies	8

^a^Based on the manufacturers’ websites, the OMEGA and M4D seem technically identical.

Typically, measuring body size using 3D scanning was a two-step process: the first step was capturing of one or multiple 2D/3D images; the second involved the postprocessing of those images to obtain a complete 3D image and derive body size parameters from that image. Postprocessing may require several steps, for which in most cases different software applications are needed. If reported, we listed the software used for postprocessing. Some studies entirely focused on postprocessing steps and deriving parameters. In one of those, a regression model was developed to calculate intracranial volume from cranial volume as measured with 3D scanning [55]. Another study used deep learning algorithms–a form of artificial intelligence–to automatically diagnose head shape deformations [39].

**Fig 6:**
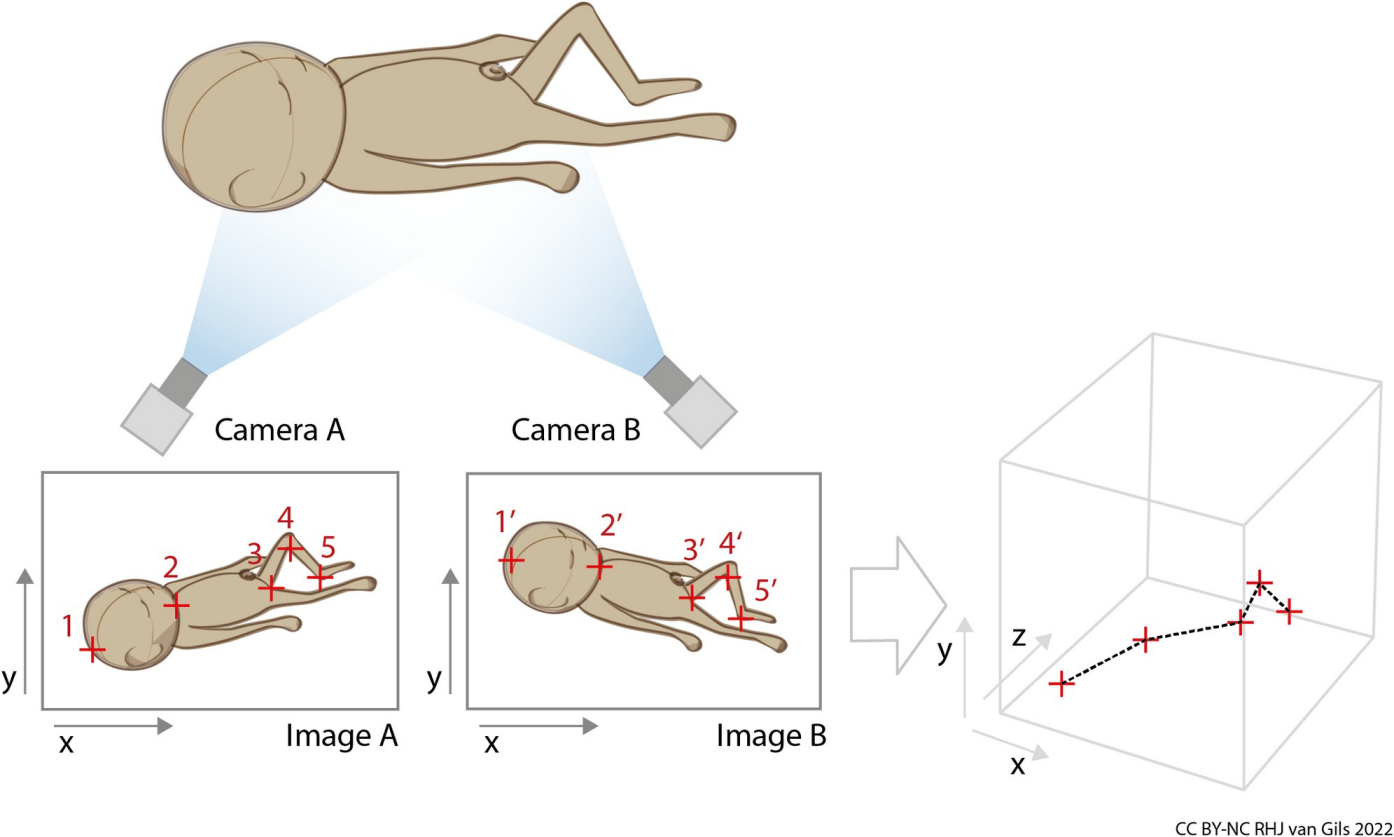
Schematic representation of the stereoscopic vision system to measure neonates’ body length.

Some studies explored novel technologies that made use of self-developed hardware and or software [34, 35, 40, 52, 53]. One of these concerned smartphone slow-motion video capture, using available (open source or freeware) photogrammetry and 3D-mesh software to obtain 360 degrees 3D scans [34]. The researchers automated the process to obtain an accurate 3D model from slow-motion video in a next study [35], thereby reducing the time and skills needed to derive measurement data. Another study used a 2D image technique via linear metric photogrammetry [56]. The technique used printed photographs to calculate body length via known dimensions of an object (for example, a table or door) depicted on the same photograph close to the person. One study evaluated a prototype with ultrasonic sensors to measure head circumference inside incubators [40]. Lastly, one study reported a self-developed stereoscopic vision system to measure body length [53] via two digital 2D images, taken from two different viewing angles (Fig 6). On both these images, the user manually places digital marks on corresponding body points–that is head-end, neck, crotch, knee, and heel–with no need to stretch the infant’s curled-up legs. From the marked body points, the stereoscopic algorithm instantly calculates the 3D-distances of corresponding body segments in mm.

Measuring cranial volume was addressed in several studies [32, 37, 43–47, 50, 51, 55]. Of those, a series of related studies [32, 37, 43], all using the STARscanner 3D scanner, aims to measure cranial volume of preterm infants as standard routine care and to develop preterm cranial volume growth reference charts [32].

### Accuracy of techniques used

The reported accuracies are presented in S3 Data column ‘reported accuracy’. Studies quantified or described accuracy in different ways. Some studies focused solely on accuracy and reliability of techniques, comparing these to a standard, routine technique, in most cases manual measurements with tape measure or calliper [33–35, 37, 38, 40–43, 45, 48–57]. In some studies, a high-resolution 3D scanner with high manufacturer-specification accuracy was used as reference for the ‘true values’, instead of manual measurements [49, 57]. Studies validating commercially available 3D scanning devices for shape or growth analysis [32, 35, 36, 39, 44, 46, 47, 54] did not perform any reference measurements to verify accuracy of scans, but relied on the manufacturers’ claimed device accuracy or previous accuracy-validating studies.

### Disturbance of patients during measuring procedures

S4 Data presents a qualitative description of the extent of disturbance. None of the studies reported sound as a disturbing factor. The most common disturbance factor was touching the patient for preparation and positioning. Nylon caps to ensure good 3D capture were needed for 3D scanning of the head [33–35, 41–44, 46–48, 50–52, 54]. The use of eye protectors was reported in some studies [33, 50]. For head measurements with a stationary 3D scanner, the infant was held by an adult or positioned in a chair [36, 37, 39, 41–47, 51, 52, 54, 55, 57], or had to lie inside the scanner during capture [32, 37, 41, 43]. For some measuring techniques, the infant had to be undressed [52, 53]. Two techniques claim no special preparation or repositioning of the patient solely for the purpose of measuring, which implies that the patient stays in bed or incubator [33, 50].

If reported, we listed the time needed to perform the actual measurement as an indicator for disturbance. Across all studies the procedure takes from 1 to 17 minutes, including preparation and the actual capture or recording time. Reported capture times ranged from 1.5 ms to 17 minutes. A few studies reported the time needed for postprocessing [35, 54], ranging from 1 to 60 minutes. However, postprocessing is a separate process that does not disturb the patient because it takes place after capture, at a computer.

### Suitability of used techniques for use in incubators

The techniques’ suitability for measuring body size of an infant lying in an incubator was assessed using the criteria earlier presented in Table 1. Techniques were classified as suitable, potential suitable or not suitable. The results of this assessment are presented in S5 Data for all included studies, with a narrative motivation per study. S6 Data summarizes the techniques judged as suitable and potentially suitable, including a motivation per criterium-score. S3 Appendix displays images of used devices in the suitable and potentially suitable techniques.

### Techniques assessed as suitable

We judged two techniques suitable for our purpose: the VECTRA H1 handheld 3D scanner [50] and a self-developed stereoscopic vision system [53].

The first suitable technique, using the Vectra 3D handheld scanner [50], involved measuring the 3D head shape of preterm infants, including cranial volume. This was applied to preterm infants, including those mechanically ventilated. Some infants (exact numbers not reported) were measured lying in an incubator, in tandem with routine care moments. Infants had to wear a cap and eye protectors against the flashlight. Ten captures per measurement and a time-consuming 3D postprocessing (49:17 ± 7:53 min.) were needed to derive 3D measurement data. Accuracy was evaluated by comparing measurements derived from the Vectra scans to high-accuracy reference 3D scans of a mannequin head, and manual measurements of six infants. This accuracy was reported as sufficient for clinical use. The interobserver reliability was not evaluated.

The second suitable technique is the stereoscopic vision system to measure body length [53] (Fig 6). Although this study did not report measuring infants lying inside an incubator, the corresponding author communicated that lab tests had been done to verify the accuracy when measuring through the incubator’s cover. Body length was measured without touching the infant and without the need to stretch the infant’s curled-up legs. The infant must be undressed during image capture. Eye protection is not needed because passive, non-photonic cameras are used. This technique gives instant measurement results after the body points have been marked, without any time needed for 3D postprocessing. Accuracy was compared to standard manual measurements and reported as clinically sufficient. The interobserver reliability was not evaluated.

### Potentially suitable techniques

Five other techniques, all using handheld 3D scanning, were classified as potentially suitable: the Scanify scanner [33, 49], OMEGA [48], M4D [54], Structure Sensor [38] and Smartphone (via slow-motion video) [34, 35]. The Scanify scanner was evaluated in a NICU-setting, measuring preterm infants lying in incubators, and met all requirements to classify as Suitable, except for accuracy. The authors state that these measurements are not yet precise enough for daily clinical use. Seventeen preterm infants lying in an incubator were involved, all with some form of respiratory aid. In total, seventy-three measurements were performed. No repositioning of the infant was reported other than placing a light shield over the infant’s eyes. Nevertheless, the projected light did cause some disturbance in some of the infants, despite the eye protection. Two separate 3D captures were needed to derive body length and head circumference with the use of a ‘digital tape measure’ to measure 2D distances on the 3D surface. The time needed to derive head circumference and body length from the 3D images was not reported.

One technique with 2D linear metric [56] could potentially be suitable, provided that a reference scale (e.g. a paper ruler) is put near the infant in the incubator, or a more advanced 2D vision technique is used capable to accurately measure without the need of reference scale. The suitability of the technique using ultrasonic sensors for measuring head circumference inside incubators [40] could not be assessed because the documentation was insufficient.

## Discussion

This systematic review aimed to identify stress-free techniques for measuring the body size of neonates and older infants that would provide alternatives to calliper and tape measure based instruments. The techniques identified here were assessed for their suitability in measuring the body size of infants lying inside incubators. As a criterion for suitability, we envisioned an ideal technique that would enable stress-free body size measurement of a ventilated, highly stress-sensitive preterm infant directly through the transparent incubator cover, without needing extra preparation or repositioning of the infant, and performed in tandem with routine care procedures. Lastly, accuracy and reliability of this ideal technique should not be user-dependent.

None of the identified techniques met all requirements of our imaginary ideal device. Two novel techniques were rated as suitable for measuring infants inside incubators, and five techniques as potentially suitable. Only two studies, one using the Scanify handheld 3D-scanner and the other the Vectra handheld 3D-scanner [33, 50], actually measured infants inside incubators. However, one or more of the incubator doors had to be opened to capture the 3D-scans, presumably because the highly reflective and curved surface of the cover could be subject to view distortions or reflections of projected light from the scanner. Furthermore, both studies with handheld 3D scanners reported problems when measuring ventilated or respiratory supported infants; the ventilation tubes with a CPAP securing hat and or gastric tubes made it difficult to capture good 3D scans and, with that, hindered deriving measurements from the 3D-images.

The aim and setting of the study using the Scanify handheld 3D-scanner to measure head circumference and body length in a NICU-setting [33] come closest to our underlying goal: enabling growth monitoring of the smallest preterm infants without causing stress. However, it was rated only as potentially suitable instead of fully suitable because the requirement for sufficient clinical accuracy was not met. Andrews and colleagues conclude that handheld 3D-scanning might be clinically suitable as a non-disturbing technique to measure body length and head circumference in a NICU-setting if its accuracy and reliability are improved. We assume that the manual process to derive these growth parameters negatively influences the accuracy and, with that, the reliability. Furthermore, this technique allows measuring the body size of an infant lying in an incubator without repositioning of the infant. The projected light did, however, disturb some of the infants, despite the eye protection. A limitation of this technique is that volumetric parameters cannot be derived. The reason for this is that the point-and-shoot capture has a limited viewing angle, and the resulting 3D images simply miss 3D data for volumetric measurements.

The technique using the Vectra 3D handheld scanner [50] was rated as suitable. The related study measured the 3D head shape of preterm infants, including cranial volume, aiming for better growth indicators than 2D head circumference. Infants were measured lying in incubators, combining 3D captures with routine care moments. The extensive process for 3D capturing and postprocessing might limit practical usability in a NICU-setting. However, this is compensated for by the high clinical value of accurate volumetric growth data of the head.

The second suitable technique is a stereoscopic vision system to measure body length [53]. The instant results, without need for time- and skill-demanding postprocessing, is a big advance over the 3D scanners. The instant results, claimed accuracy, and the relatively cheap hardware makes this system feasible for body length measurements in a NICU-setting. Nevertheless, placing the digital marks on the body points on the images could compromise the inter-observer reliability of this system. The biggest advance of this technique is that stretching the infant’s legs is no longer necessary. However, the infant must be undressed, which is potentially disturbing. But this disturbance could be minimized if image capture is combined with a routine diaper change. On the other hand, eye protection is not needed because the technique uses passive, non-photonic cameras. Still, the low-light NICU-conditions might need additional light; it is unknown if low-light conditions were simulated in the lab test, in which objects inside an incubator were measured through the transparent cover.

3D scanning is clearly dominant, as it is used in 23 out of the 26 studies identified. The majority of those studies concerned head measurements. The dominance of 3D scanning could be related to the growing consensus that volumetric 3D body size parameters are more predictive for physical growth and brain development than the current 2D parameters body length and head circumference. Multiple studies have concluded that the 3D cranial volume is a more accurate predictor for brain development than is head circumference, because infants with equal head circumference may differ in cranial volume [37, 43, 45, 47, 50]. Several studies have focussed on techniques to measure cranial volume, and growth reference charts for cranial volume in late preterm and term neonates have been developed recently [32].

Although 3D scanning seems to meet all requirements for accurate and contactless measuring, the majority of studies exposed barriers to the practical usability of 3D scanning in routine NICU care. Those barriers relate to both the capturing and the postprocessing of 3D data. The extensive ‘manual’ steps with, in most cases, multiple software applications highly influence the accuracy of measurements. These postprocessing steps not only require advanced 3D software skills, but also clinical knowledge to accurately mark anthropometrical points on the 3D images. One study reported postprocessing times from 45 to 60 minutes per scan [54]. Another study preferred traditional manual techniques over 3D scanning because the manual measurements were significantly faster, and 3D did not improve accuracy [48]. To limit user influence on accuracy, automation of postprocessing might be the biggest challenge to developing accurate 3D scanning techniques for routine care. An example is a further study of the smartphone-based 3D photogrammetry technique, which solely focused on automation of 3D postprocessing to improve non-expert intra- and inter-user accuracy [35]. The fast-upcoming field of artificial intelligence-based technologies for image manipulation and recognition might contribute to 3D postprocessing automation, especially deriving measurement data from 3D images. Remarkably, artificial intelligence was involved in only one study of this review [39], and the authors of another study suggested that artificial intelligence could be used in future to automate manual steps [53].

The aim and design of this review, as well as the data analysis of included studies, may be subject to limitations. First, large differences between studies in settings and aims hindered extracting uniform data for comparing and assessing techniques. For example, the accuracy of a technique, in clinical practice, is a result of the (manufacturer claimed) device-related instrument accuracy and the user-actions that influence measurement outcomes. In included studies, the techniques’ accuracy was reported in varies ways, limiting comparison and assessment. Second, the possibly interpretative, subjective expert judgement used to assess suitability of techniques for measuring infants inside incubators might compromise the reproducibility of our findings. However, we tried to avoid monodisciplinary judgement by combining reviewers’ judgements from a clinical and technical perspective. Lastly, only scientific databases were searched to identify techniques. However, an industry patent search for clinical body size measuring techniques was done parallel to this review, but not reported here. Furthermore, we limited our eligibility criteria to techniques already used in a clinical setting. In both science and industry, technologies in the field of 3D machine and computer vision are being developed for non-clinical size measurements, for applications such as fashion, face recognition, industrial manufacturing, and the agricultural and livestock industry. However, making such non-clinical techniques suitable for a NICU-setting is a very complex and uncertain route.

Other systematic reviews identifying body size measuring techniques for neonates or older infants were not available at the time of writing this review. A few studies included in this review focused on our underlying goal as well: the pursuit of minimal-disturbing techniques for growth monitoring of preterm infants lying in incubators, even when they are sick and unstable [33, 40, 50, 53].

## Conclusions

The trend to replace 2D manual body size measuring techniques with 3D scanning cannot be denied. Volumetric 3D body size parameters are clinically superior to traditional 2D parameters, enabling more accurate growth monitoring. Growth reference charts based on volumetric parameters, such as cranial volume, could be the new standard in the future. In addition, 3D measurements can be contactless, enabling stress-free growth monitoring of even the smallest preterm infants. However, the complex, time-consuming 3D postprocessing challenges the clinical and practical NICU-usability of 3D techniques. Regrettably, none of the identified suitable 3D techniques met all our requirements of an ideal all-in-one body size measuring technique for extreme preterm infants. For future research, a first step in developing this ideal technique could be devising a head scanner that enables cranial volume measurements of infants lying in incubators. Handheld 3D scanning with a compact device may have good properties in this respect. Handheld or other technique, this device should capture a 360 degrees 3D image of the head, with sufficient accuracy for clinical use, and with all the practical restrictions of the NICU-environment taken into account: image capture from outside the incubator, breathing tubes blocking the view, low light conditions, and not touching the infant solely for the measurement. Furthermore, a literature search beyond the boundaries of our medical databases, into studies or industry developments in the field of 2D and 3D vision technologies, might find the ideal suitable technique not identified in this systematic review.

## Supporting information

S1 Checklist PRISMA 2020 checklist. (PDF)

S1 Appendix Search syntax of electronic database search with the number of records identified per database. (PDF)

S2 Appendix General characteristics to categorize 3D scanning techniques. (PDF)

S3 Appendix Images of devices of suitable and potentially suitable techniques. (PDF)

S1 Data All-data-table containing all extracted data of included studies. (XLSX)

S2 Data Characteristics and quality assessment. (PDF)

S3 Data Body size measurement technology type. (PDF)

S4 Data Disturbance of patients during measurement. (PDF)

S5 Data Assessment of the techniques’ suitability for measuring the body size of preterm infants lying in an incubator. (PDF)

S6 Data Techniques assessed as suitable or potentially suitable for measuring body size of preterm infants lying in incubators. (PDF)

## References

[1] Ong KK, Kennedy K, Castañeda-Gutiérrez E, Forsyth S, Godfrey KM, Koletzko B, et al. Postnatal growth in preterm infants and later health outcomes: a systematic review. Acta Paediatr. 2015;104(10):974–86. Epub 2015/07/17. doi: 10.1111/apa.13128 ; PubMed Central PMCID: PMC5054880. 26179961 PMC5054880

[2] Strydom K, Van Niekerk E, Dhansay MA. Factors affecting body composition in preterm infants: Assessment techniques and nutritional interventions. Pediatr Neonatol. 2019;60(2):121–8. Epub 2017/12/15. doi: 10.1016/j.pedneo.2017.10.007 . 29239827

[3] Greer FR, Olsen IE. How Fast Should the Preterm Infant Grow? Current Pediatrics Reports. 2013;1(4):240–6. doi: 10.1007/s40124-013-0029-1

[4] Andrews ET, Ashton JJ, Pearson F, Beattie RM, Johnson MJ. Early postnatal growth failure in preterm infants is not inevitable. Arch Dis Child Fetal Neonatal Ed. 2018. Epub 2018/08/24. doi: 10.1007/s10815-018-1209-2 . 30135111

[5] Villar J, Giuliani F, Bhutta ZA, Bertino E, Ohuma EO, Ismail LC, et al. Postnatal growth standards for preterm infants: the Preterm Postnatal Follow-up Study of the INTERGROWTH-21(st) Project. Lancet Glob Health. 2015;3(11):e681–91. Epub 2015/10/18. doi: 10.1016/S2214-109X(15)00163-1 . 26475015

[6] Villar J, Altman DG, Purwar M, Noble JA, Knight HE, Ruyan P, et al. The objectives, design and implementation of the INTERGROWTH-21st Project. Bjog. 2013;120 Suppl 2:9–26, v. Epub 2013/05/18. doi: 10.1111/1471-0528.12047 . 23678873

[7] Ranger M, Grunau RE. Early repetitive pain in preterm infants in relation to the developing brain. Pain Manag. 2014;4(1):57–67. Epub 2014/03/20. doi: 10.2217/pmt.13.61 ; PubMed Central PMCID: PMC3975052. 24641344 PMC3975052

[8] Brummelte S, Grunau RE, Chau V, Poskitt KJ, Brant R, Vinall J, et al. Procedural pain and brain development in premature newborns. Ann Neurol. 2012;71(3):385–96. Epub 2012/03/01. doi: 10.1002/ana.22267 ; PubMed Central PMCID: PMC3760843. 22374882 PMC3760843

[9] Ranger M, Chau CM, Garg A, Woodward TS, Beg MF, Bjornson B, et al. Neonatal pain-related stress predicts cortical thickness at age 7 years in children born very preterm. PLoS One. 2013;8(10):e76702. Epub 2013/11/10. doi: 10.1371/journal.pone.0076702 ; PubMed Central PMCID: PMC3800011. 24204657 PMC3800011

[10] Grunau RE. Neonatal pain in very preterm infants: long-term effects on brain, neurodevelopment and pain reactivity. Rambam Maimonides Med J. 2013;4(4):e0025. Epub 2013/11/15. doi: 10.5041/RMMJ.10132 ; PubMed Central PMCID: PMC3820298. 24228168 PMC3820298

[11] Doesburg SM, Chau CM, Cheung TPL, Moiseev A, Ribary U, Herdman AT, et al. Neonatal pain-related stress, functional cortical activity and visual-perceptual abilities in school-age children born at extremely low gestational age. Pain. 2013;154(10):1946–52. Epub 2013/05/29. doi: 10.1016/j.pain.2013.04.009 ; PubMed Central PMCID: PMC3778166. 23711638 PMC3778166

[12] Grunau RE, Whitfield MF, Petrie-Thomas J, Synnes AR, Cepeda IL, Keidar A, et al. Neonatal pain, parenting stress and interaction, in relation to cognitive and motor development at 8 and 18 months in preterm infants. Pain. 2009;143(1–2):138–46. Epub 2009/03/25. doi: 10.1016/j.pain.2009.02.014 ; PubMed Central PMCID: PMC2836793. 19307058 PMC2836793

[13] Ranger M, Zwicker JG, Chau CM, Park MT, Chakravarthy MM, Poskitt K, et al. Neonatal Pain and Infection Relate to Smaller Cerebellum in Very Preterm Children at School Age. J Pediatr. 2015;167(2):292–8.e1. Epub 2015/05/20. doi: 10.1016/j.jpeds.2015.04.055 . 25987534

[14] Kaneshi Y, Ohta H, Morioka K, Hayasaka I, Uzuki Y, Akimoto T, et al. Influence of light exposure at nighttime on sleep development and body growth of preterm infants. Scientific reports. 2016;6:21680. doi: 10.1038/srep21680 26877166 PMC4753683

[15] Cardoso SM, Kozlowski Lde C, Lacerda AB, Marques JM, Ribas A. Newborn physiological responses to noise in the neonatal unit. Braz J Otorhinolaryngol. 2015;81(6):583–8. doi: 10.1016/j.bjorl.2014.11.008 . 26480903 PMC9442682

[16] Kuhn P, Zores C, Langlet C, Escande B, Astruc D, Dufour A. Moderate acoustic changes can disrupt the sleep of very preterm infants in their incubators. Acta Paediatr. 2013;102(10):949–54. doi: 10.1111/apa.12330 . 23800026

[17] Kuhn P, Zores C, Pebayle T, Hoeft A, Langlet C, Escande B, et al. Infants born very preterm react to variations of the acoustic environment in their incubator from a minimum signal-to-noise ratio threshold of 5 to 10 dBA. Pediatr Res. 2012;71(4 Pt 1):386–92. doi: 10.1038/pr.2011.76 . 22391640

[18] Brown G. NICU Noise and the Preterm Infant. Neonatal Network. 2009;28(3):165–73. doi: 10.1891/0730-0832.28.3.165 19451078

[19] Korotchikova I, Stevenson NJ, Livingstone V, Ryan CA, Boylan GB. Sleep-wake cycle of the healthy term newborn infant in the immediate postnatal period. Clin Neurophysiol. 2016;127(4):2095–101. doi: 10.1016/j.clinph.2015.12.015 . 26790580

[20] Bueno C, Menna-Barreto L. Development of sleep/wake, activity and temperature rhythms in newborns maintained in a neonatal intensive care unit and the impact of feeding schedules. Infant Behav Dev. 2016;44:21–8. doi: 10.1016/j.infbeh.2016.05.004 . 27261553

[21] Moody C, Callahan TJ, Aldrich H, Gance-Cleveland B, Sables-Baus S. Early Initiation of Newborn Individualized Developmental Care and Assessment Program (NIDCAP) Reduces Length of Stay: A Quality Improvement Project. J Pediatr Nurs. 2017;32:59–63. Epub 2016/12/08. doi: 10.1016/j.pedn.2016.11.001 . 27923536

[22] Als H, Duffy FH, McAnulty G, Butler SC, Lightbody L, Kosta S, et al. NIDCAP improves brain function and structure in preterm infants with severe intrauterine growth restriction. J Perinatol. 2012;32(10):797–803. doi: 10.1038/jp.2011.201 ; PubMed Central PMCID: PMC3461405. 22301525 PMC3461405

[23] VandenBerg KA. Individualized developmental care for high risk newborns in the NICU: A practice guideline. Early Human Development. 2007;83(7):433–42. doi: 10.1016/j.earlhumdev.2007.03.008 17467932

[24] Pereira-Da-Silva L, Bergmans KIM, van Kerkhoven LAS, Leal F, Virella D, Videira-Amaral JM. Reducing discomfort while measuring crown-heel length in neonates. Acta Paediatrica (Oslo, Norway: 1992). 2006;95(6):742–6. doi: 10.1080/08035250500516623 . 16754558

[25] Lawn CJ, Chavasse RJ, Booth KA, Angeles M, Weir FJ. The neorule: a new instrument to measure linear growth in preterm infants. Arch Dis Child Fetal Neonatal Ed. 2004;89(4):F360–3. Epub 2004/06/24. doi: 10.1136/adc.2002.019448 ; PubMed Central PMCID: PMC1721729. 15210676 PMC1721729

[26] Sell E. Een nieuwe lengtemeting bij neonaten: Een praktijkonderzoek naar de haalbaarheid van een prototype babylengtemeter [Bachelor Thesis]. 15-06-2017: Rotterdam University of Applied Sciences; 2017.

[27] Meulen Rvd. Schedelomtrek meten bij kinderen op een Intensive Care Neonatologie, post Intensive Care en High Care Neonatologie [Bachelor Thesis]: Rotterdam University of Applied Sciences; 2017.

[28] Page MJ, McKenzie JE, Bossuyt PM, Boutron I, Hoffmann TC, Mulrow CD, et al. The PRISMA 2020 statement: an updated guideline for reporting systematic reviews. BMJ. 2021;372:n71. doi: 10.1136/bmj.n71 33782057 PMC8005924

[29] Bartram JL, Rigby AS, Baxter PS. The "Lasso-o" tape: Stretchability and observer variability in head circumference measurement. Arch Dis Child. 2005;90(8):820–1. doi: 10.1136/adc.2004.063743 16040879 PMC1720542

[30] Kmet LM, Cook LS, Lee RC. Standard quality assessment criteria for evaluating primary research papers from a variety of fields. 2004.

[31] Villar J, Giuliani F, Fenton TR, Ohuma EO, Ismail LC, Kennedy SH. INTERGROWTH-21st very preterm size at birth reference charts. The Lancet. 2016;387(10021):844–5. doi: 10.1016/S0140-6736(16)00384-6 26898853

[32] Vermeulen MJ, Burkhardt W, Fritze A, Roelants J, Mense L, Willemsen S, et al. Reference Charts for Neonatal Cranial Volume Based on 3D Laser Scanning to Monitor Head Growth. Front Pediatr. 2021;9. doi: 10.3389/fped.2021.654112 34123964 PMC8192695

[33] Andrews ET, Ashton JJ, Pearson F, Beattie RM, Johnson MJ. Handheld 3D scanning as a minimally invasive measuring technique for neonatal anthropometry. Clin Nutr ESPEN. 2019;33:279–82. doi: 10.1016/j.clnesp.2019.06.012 31451267

[34] Barbero-García I, Lerma JL, Marqués-Mateu Á, Miranda P. Low-Cost Smartphone-Based Photogrammetry for the Analysis of Cranial Deformation in Infants. World Neurosurgery. 2017;102:545–54. doi: 10.1016/j.wneu.2017.03.015 28300713

[35] Barbero-García I, Lerma JL, Mora-Navarro G. Fully automatic smartphone-based photogrammetric 3D modelling of infant’s heads for cranial deformation analysis. ISPRS Journal of Photogrammetry and Remote Sensing. 2020;166:268–77. 10.1016/j.isprsjprs.2020.06.013.

[36] Brons S, Meulstee JW, Nada RM, Kuijpers MAR, Bronkhorst EM, Berge SJ, et al. Uniform 3D meshes to establish normative facial averages of healthy infants during the first year of life. PLoS ONE. 2019;14(5):e0217267. doi: 10.1371/journal.pone.0217267 . 31107914 PMC6527206

[37] Burkhardt W, Schneider D, Hahn G, Konstantelos D, Maas HG, Rüdiger M. Non-invasive estimation of brain-volume in infants. Early Hum Dev. 2019;132:52–7. doi: 10.1016/j.earlhumdev.2019.03.020 30986647

[38] Conkle J, Keirsey K, Hughes A, Breiman J, Ramakrishnan U, Suchdev PS, et al. A collaborative, mixed-methods evaluation of a low-cost, handheld 3D imaging system for child anthropometry. Matern Child Nutr. 2019;15(2):e12686–e. Epub 2018/10/18. doi: 10.1111/mcn.12686 . 30194911 PMC6519116

[39] de Jong G, Bijlsma E, Meulstee J, Wennen M, van Lindert E, Maal T, et al. Combining deep learning with 3D stereophotogrammetry for craniosynostosis diagnosis. Sci Rep. 2020;10(1):15346. doi: 10.1038/s41598-020-72143-y . 32948813 PMC7501225

[40] Firmansyah R, Widodo A, Romadhon AD, Hudha MS, Saputra PPS, Lestari NA. The prototype of infant incubator monitoring system based on the internet of things using NodeMCU ESP8266. National Physics Seminar. Journal of Physics Conference Series. 1171. Bristol: Iop Publishing Ltd; 2019.

[41] Geil MD, Smith A. Accuracy and Reliability of a System for the Digital Capture of Infant Head Shapes in the Treatment of Cranial Deformities. JPO: Journal of Prosthetics and Orthotics. 2008;20(2).

[42] Goto L, Lee W, Molenbroek JFM, Cabo AJ, Goossens RHM. Traditional and 3D scan extracted measurements of the heads and faces of Dutch children. International Journal of Industrial Ergonomics. 2019;73:102828. 10.1016/j.ergon.2019.102828.

[43] Ifflaender S, Rüdiger M, Koch A, Burkhardt W. Three-Dimensional Digital Capture of Head Size in Neonates—A Method Evaluation. PLoS ONE. 2013;8(4). doi: 10.1371/journal.pone.0061274 23580107 PMC3620274

[44] Linz C, Meyer-Marcotty P, Böhm H, Müller-Richter U, Jager B, Hartmann S, et al. 3D stereophotogrammetric analysis of operative effects after broad median craniectomy in premature sagittal craniosynostosis. Child’s Nerv Syst. 2014;30(2):313–8. doi: 10.1007/s00381-013-2253-y 23955177

[45] Martini M, Klausing A, Lüchters G, Heim N, Messing-Jünger M. Head circumference—a useful single parameter for skull volume development in cranial growth analysis? Head Face Med. 2018;14(1):3. doi: 10.1186/s13005-017-0159-8 29321071 PMC5764008

[46] Meyer-Marcotty P, Böhm H, Linz C, Kochel J, Stellzig-Eisenhauer A, Schweitzer T. Three-dimensional analysis of cranial growth from 6 to 12 months of age. Eur J Orthod. 2014;36(5):489–96. doi: 10.1093/ejo/cjt010 25257925

[47] Meyer-Marcotty P, Kunz F, Schweitzer T, Wachter B, Bohm H, Wasmuth N, et al. Cranial growth in infants-A longitudinal three-dimensional analysis of the first months of life. J Craniomaxillofac Surg. 2018;46(6):987–93. doi: 10.1016/j.jcms.2018.04.009 . 29709329

[48] Nahles S, Klein M, Yacoub A, Neyer J. Evaluation of positional plagiocephaly: Conventional anthropometric measurement versus laser scanning method. J Craniomaxillofac Surg. 2018;46(1):11–21. Epub 2017/11/16. doi: 10.1016/j.jcms.2017.10.010 . 29137851

[49] Ritschl LM, Roth M, Fichter AM, Mittermeier F, Kuschel B, Wolff KD, et al. The possibilities of a portable low-budget three-dimensional stereophotogrammetry system in neonates: a prospective growth analysis and analysis of accuracy. Head Face Med. 2018;14(1):11. Epub 2018/08/05. doi: 10.1186/s13005-018-0168-2 ; PubMed Central PMCID: PMC6076401. 30075821 PMC6076401

[50] Santander P, Quast A, Hubbert J, Horn S, Meyer-Marcotty P, Kuster H, et al. Three-dimensional head shape acquisition in preterm infants—Translating an orthodontic imaging procedure into neonatal care. Early Hum Dev. 2019;140:104908. doi: 10.1016/j.earlhumdev.2019.104908 . 31670175

[51] Schaaf H, Pons-Kuehnemann J, Malik CY, Streckbein P, Preuss M, Howaldt HP, et al. Accuracy of three-dimensional photogrammetric images in non-synostotic cranial deformities. Neuropediatrics. 2010;41(1):24–9. Epub 2010/06/24. doi: 10.1055/s-0030-1255060 . 20571987

[52] Schloesser RL, Lauff M, Buxmann H, Veit K, Fischer D, Allendorf A. Three-dimensional body scanning: A new method to estimate body surface area in neonates. Neonatology. 2011;100(3):260–4. doi: 10.1159/000327516 21701216

[53] Sokolover N, Phillip M, Sirota L, Potruch A, Kiryati N, Klinger G, et al. A novel technique for infant length measurement based on stereoscopic vision. Arch Dis Child Educ Pract Ed. 2014;99(7):625–8. doi: 10.1136/archdischild-2013-304291 24534816

[54] Tenhagen M, Bruse JL, Rodriguez-Florez N, Angullia F, Borghi A, Koudstaal MJ, et al. Three-Dimensional Handheld Scanning to Quantify Head-Shape Changes in Spring-Assisted Surgery for Sagittal Craniosynostosis. J Craniofac Surg. 2016;27(8):2117–23. Epub 2016/12/23. doi: 10.1097/SCS.0000000000003108 . 28005766

[55] Tu LY, Porras AR, Enquobahrie A, Buck GC, Tsering D, Horvath S, et al. Automated Measurement of Intracranial Volume Using Three-Dimensional Photography. Plastic and Reconstructive Surgery. 2020;146(3):314E–23E. doi: 10.1097/PRS.0000000000007066 . 32459727

[56] Wang JC, Chang WP, Wang D, Guo HR. The feasibility of using photographs to estimate historical heights of children. Acta Paediatr Taiwan. 2000;41(3):136–9. 10920546

[57] Weinberg SM, Naidoo S, Govier DP, Martin RA, Kane AA, Marazita ML. Anthropometric precision and accuracy of digital three-dimensional photogrammetry: comparing the Genex and 3dMD imaging systems with one another and with direct anthropometry. J Craniofac Surg. 2006;17(3):477–83. Epub 2006/06/14. doi: 10.1097/00001665-200605000-00015 . 16770184

[58] Bartels J. White Paper 3D Technologies. [White Paper]. In press 2016.

