# Supplementary material for: Body size measuring techniques enabling stress-free growth monitoring of extreme preterm infants inside incubators: A systematic review
Source: PLoS One. 2022 Apr 22;17(4):e0267285. doi: 10.1371/journal.pone.0267285 (PMC9033282; doi:10.1371/journal.pone.0267285)
Supplement: S1 Appendix — (PDF) [file pone.0267285.s002.pdf]

Search syntaxes of electronic database search with the number of records identified per database

Search strategies developed and updated by the Erasmus MC Medical Library.

16-07-2021:

Search ID: 20210716 Ronald van Gils

premature body size measurement

Database searched	Platform	Years of coverage	Records	Records after duplicates removed
Embase	Embase.com	1971 - Present	3786	3716
Medline ALL	Ovid	1946 - Present	2886	1184
Web of Science Core Collection*	Web of Knowledge	1975 - Present	1926	743
Cochrane Central Register of Controlled Trials	Wiley	1992 - Present	957	629
**Total**			**9555**	**6272**

*Science Citation Index Expanded (1975-present) ; Social Sciences Citation Index (1975-present) ; Arts & Humanities Citation Index (1975-present) ; Conference Proceedings Citation Index- Science (1990-present) ; Conference Proceedings Citation Index- Social Science & Humanities (1990-present) ; Emerging Sources Citation Index (2005-present)

Embase.com (1971-)

('prematurity'/exp OR 'low birth weight'/exp OR 'incubator'/exp OR (infant/de AND ('growth, development and aging'/de OR growth/de)) OR 'child growth'/de OR (((prematur* OR preterm* OR pre-term* OR vlbw OR elbw OR lbw OR sga) NEAR/6 (birth OR childbirth OR born OR newborn* OR new*-born* OR neonat* OR infan* OR child*)) OR (small NEXT/3 (date OR gestation*)) OR (low NEAR/3 (birth-weight OR birthweight)) OR (born NEAR/3 (before OR 37) NEAR/3 week*) OR (weigh* NEAR/3 (1000 OR 1500 OR 2000 OR 1-000 OR 1-500 OR 2-000) NEAR/3 (gram* OR g) NEAR/3 birth) OR incubator* OR ((small* OR growth) NEAR/3 (newborn* OR neonat* OR infant*))):ab,ti OR (((growth OR develop*) NEAR/3 (month*) NEAR/3 age) OR ((child* OR infant*) NEXT/1 growth)):ti) AND ('body size'/exp OR 'body height'/de OR 'head circumference'/de OR 'crown rump length'/de OR 'chest circumference'/de OR 'leg length'/de OR anthropometry/de OR 'morphometry'/de OR cephalometry/de OR ('organ size'/de AND ('foot'/de OR 'lower limb'/de OR leg/de)) OR 'arm circumference'/de OR 'thigh circumference'/de OR 'foot length'/de OR (((body OR foot OR leg OR limb* OR extremit* OR knee-heel OR heel-knee OR crown-heel) NEAR/3 (size OR length OR height

OR surface*)) OR ((head OR skull OR crani* OR rump OR chest OR Mid-arm OR calf OR thigh) NEAR/3 (circumferen* OR growth)) OR anthropometr* OR cephalometr* OR morphometr* OR (growth NEAR/3 velocit*)):ab,ti OR (length NEAR/3 measurement*):ti) AND (measurement/mj OR 'measurement accuracy'/de OR 'measurement repeatability'/de OR 'measurement precision'/de OR 'reliability'/de OR 'procedures'/de OR 'validation process'/de OR 'validation study'/de OR 'observer variation'/de OR reproducibility/de OR devices/de OR ((measur* NEAR/6 (method* OR device* OR technique* OR direct* OR indirect* OR accuracy OR accurate OR precision* OR evaluat* OR compar* OR performan* OR system*)) OR repeatab* OR reliab* OR tool OR tools OR instrument* OR procedure* OR validat* OR validit* OR verif* OR ((measurement* OR measuring) NEAR/3 (size OR length OR weight OR circumferen*)) OR ((handling OR technical) NEAR/3 error*) OR ((observer* OR interobserver* OR intraobserver*) NEAR/3 (varia*))):ab,ti OR (method* OR (length NEAR/3 (measurement* OR neonat* OR newborn*))):ti) NOT ([animals]/lim NOT [humans]/lim)

Medline ALL Ovid (1946-)

(exp Infant, Premature / OR exp Infant, Low Birth Weight / OR Incubators, Infant / OR (exp Infant/ AND (Growth and Development / OR Growth/)) OR (((prematur* OR preterm* OR pre-term* OR vlbw OR elbw OR lbw OR sga) ADJ6 (birth OR childbirth OR born OR newborn* OR new*-born* OR neonat* OR infan* OR child*)) OR (small ADJ3 (date OR gestation*)) OR (low ADJ3 (birth-weight OR birthweight)) OR (born ADJ3 (before OR 37) ADJ3 week*) OR (weigh* ADJ3 (1000 OR 1500 OR 2000 OR 1-000 OR 1-500 OR 2-000) ADJ3 (gram* OR g) ADJ3 birth) OR incubator* *OR ((small* OR growth) ADJ3 (newborn* OR neonat* OR infant*))*).ab,ti. OR (((growth OR develop*) ADJ3 (month*) ADJ3 age) OR ((child* OR infant*) ADJ growth)).ti.) AND (Body Size/ OR Body Height/ OR Crown-Rump Length/ OR Anthropometry/ OR morphometry/ OR Cephalometry/ OR (Organ Size/ AND (Foot/ OR Lower Extremity/ OR Leg/)) OR (((body OR foot OR leg OR limb* OR extremit* OR knee-heel OR heel-knee OR crown-heel) ADJ3 (size OR length OR height OR surface*)) OR ((head OR skull OR crani* OR rump OR chest OR Mid-arm OR calf OR thigh) ADJ3 (circumferen* OR growth)) OR anthropometr* OR cephalometr* OR morphometr* OR (growth ADJ3 velocit*)).ab,ti. OR (length ADJ3 measurement*).ti.) AND (Dimensional Measurement Accuracy/ OR Reproducibility of Results/ OR Methods/ OR Methods.fs. OR instrumentation.fs. OR Validation Study.pt. OR Validation Studies as Topic/ OR Observer Variation/ OR "Equipment and Supplies"/ OR ((measur* ADJ6 (method* OR device* OR technique* OR direct* OR indirect* OR accuracy OR accurate OR precision* OR evaluat* OR compar* OR performan* OR system*)) OR repeatab* OR reliab* OR tool OR tools OR instrument* OR procedure* OR validat* OR validit* OR verif* OR ((measurement* OR measuring) ADJ3 (size OR length OR weight OR circumferen*)) OR ((handling OR technical) ADJ3 error*) OR ((observer* OR interobserver* OR intraobserver*) ADJ3 (varia*))).ab,ti. OR (method* OR (length ADJ3 (measurement* OR neonat* OR newborn*))).ti.) NOT (exp animals/ NOT humans/)

Web of Science Core Collection (1975-)

((TS=(((prematur* OR preterm* OR pre-term* OR vlbw OR elbw OR lbw OR sga) NEAR/5 (birth OR childbirth OR born OR newborn* OR "new* born*" OR neonat* OR infan* OR child*)) OR (small NEAR/2 (date OR gestation*)) OR (low NEAR/2 (birth-weight OR birthweight)) OR (born NEAR/2 (before) NEAR/2 week*) OR incubator* OR ((small* OR growth) NEAR/2 (newborn* OR neonat* OR infant*))) OR TI=(((growth OR develop*) NEAR/2 (month*) NEAR/2 age) OR ((child* OR infant*) NEAR/1 growth))) AND (TS=(((body OR foot OR leg OR limb* OR extremit* OR knee-heel OR heel-knee OR crown-heel) NEAR/2 (size OR length OR height OR surface*)) OR ((head OR skull OR crani* OR rump OR chest OR Mid-arm OR calf OR thigh) NEAR/2 (circumferen* OR growth)) OR anthropometr* OR cephalometr* OR morphometr* OR (growth NEAR/2 velocit*)) OR TI=(length NEAR/2 measurement*)) AND (TS=((measur* NEAR/5 (method* OR device* OR technique* OR

direct* OR indirect* OR accuracy OR accurate OR precision* OR evaluat* OR compar* OR performan* OR system*)) OR repeatab* OR reliab* OR tool OR tools OR instrument* OR procedure* OR validat* OR validit* OR verif* OR ((measurement* OR measuring) NEAR/2 (size OR length OR weight OR circumferen*)) OR ((handling OR technical) NEAR/2 error*) OR ((observer* OR interobserver* OR intraobserver*) NEAR/2 (varia*))) OR TI=(method* OR (length NEAR/2 (measurement* OR neonat* OR newborn*)))))

Cochrane CENTRAL register of trials (1992-)

((((prematur* OR preterm* OR pre NEXT term* OR vlbw OR elbw OR lbw OR sga) NEAR/6 (birth OR childbirth OR born OR newborn* OR "new* born*" OR neonat* OR infan* OR child*)) OR (small NEXT/3 (date OR gestation*)) OR (low NEAR/3 (birth NEXT weight OR birthweight)) OR (born NEAR/3 (before) NEAR/3 week*) OR incubator* OR ((small* OR growth) NEAR/3 (newborn* OR neonat* OR infant*))):ab,ti OR (((growth OR develop*) NEAR/3 (month*) NEAR/3 age) OR ((child* OR infant*) NEXT/1 growth)):ti) AND ((((body OR foot OR leg OR limb* OR extremit* OR knee NEXT heel OR heel NEXT knee OR crown NEXT heel) NEAR/3 (size OR length OR height OR surface*)) OR ((head OR skull OR crani* OR rump OR chest OR Mid NEXT arm OR calf OR thigh) NEAR/3 (circumferen* OR growth)) OR anthropometr* OR cephalometr* OR morphometr* OR (growth NEAR/3 velocit*)):ab,ti OR (length NEAR/3 measurement*):ti) AND (((measur* NEAR/6 (method* OR device* OR technique* OR direct* OR indirect* OR accuracy OR accurate OR precision* OR evaluat* OR compar* OR performan* OR system*)) OR repeatab* OR reliab* OR tool OR tools OR instrument* OR procedure* OR validat* OR validit* OR verif* OR ((measurement* OR measuring) NEAR/3 (size OR length OR weight OR circumferen*)) OR ((handling OR technical) NEAR/3 error*) OR ((observer* OR interobserver* OR intraobserver*) NEAR/3 (varia*))):ab,ti OR (method* OR (length NEAR/3 (measurement* OR neonat* OR newborn*))):ti)
