# Supplementary material for: Body size measuring techniques enabling stress-free growth monitoring of extreme preterm infants inside incubators: A systematic review
Source: PLoS One. 2022 Apr 22;17(4):e0267285. doi: 10.1371/journal.pone.0267285 (PMC9033282; doi:10.1371/journal.pone.0267285)
Supplement: S2 Appendix — (PDF) [file pone.0267285.s003.pdf]

General characteristics to categorize 3D scanning techniques

Characteristic	Description
Photogrammetry, including stereoscopic vision	Photogrammetry uses **two or more 2D images taken from different viewing angles to calculate 3D information via corresponding points of an object on both images and the relative positions from which the images are captured** (Fig 1 and 2). This also includes 'structure from motion', using video capture to obtain multiple images from different viewing angles. These techniques can be referred to as 'non-photonic' (also referred to as 'passive') meaning that no additional light is used other than existing ambient light to capture the images. However, sufficient ambient light is needed to capture images with sufficient detail.
Structured light, including laser triangulation	Projects a beam of structured light, as point grid, lines, fringes, on an object. The projected light is deformed by the shape of this object. A camera, under an angle with the projected light, captures a 2D image of the object and deformed light. **3D depth is calculated based on the deformation of projected light** (Fig 3). Because these techniques need some form of additional light (in the visual or infrared spectrum) to capture images, they can be referred to as 'photonic' scanning techniques (also referred to as 'active'). Some photogrammetry scanners use additional structured light as extra source for depth calculation. Photogrammetry scanners that use additional flashlight to capture sufficiently lighted 2D images, but not needed to calculate 3D depth, are also listed as photonic.

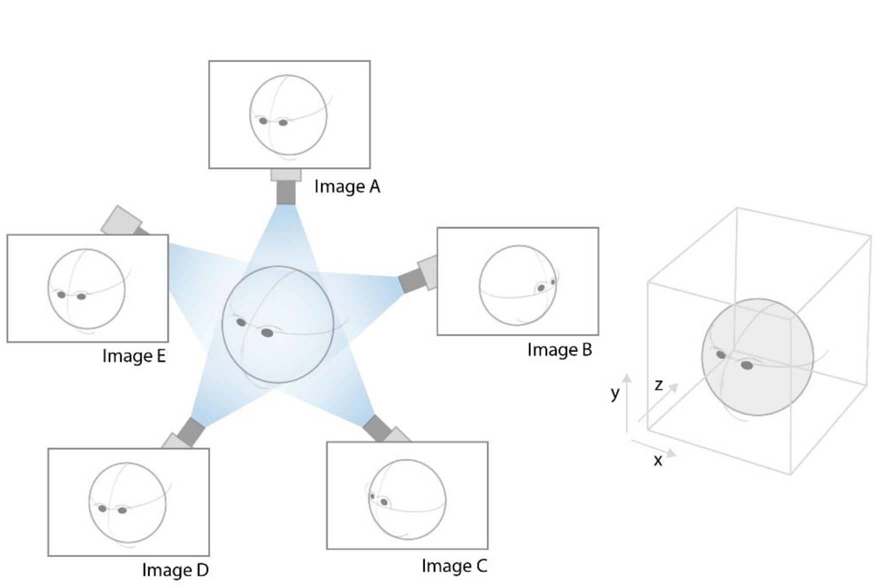

Fig 1. Photogrammetry uses multiple images from different viewing angles to derive depth information of an object. Images can be captures by one camera, moving the camera or object to a different position, or with multiple cameras in different position around the object. A 360 degrees 3D scan is possible when images are captured around the object.

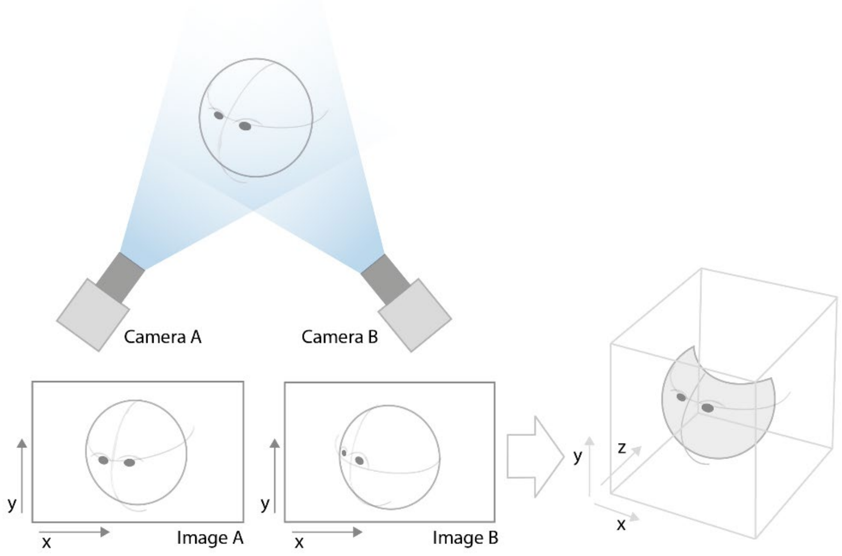

Fig 2. Stereoscopic vision uses two cameras to derive depth information. The maximum area is limited by the viewing angles of the two cameras

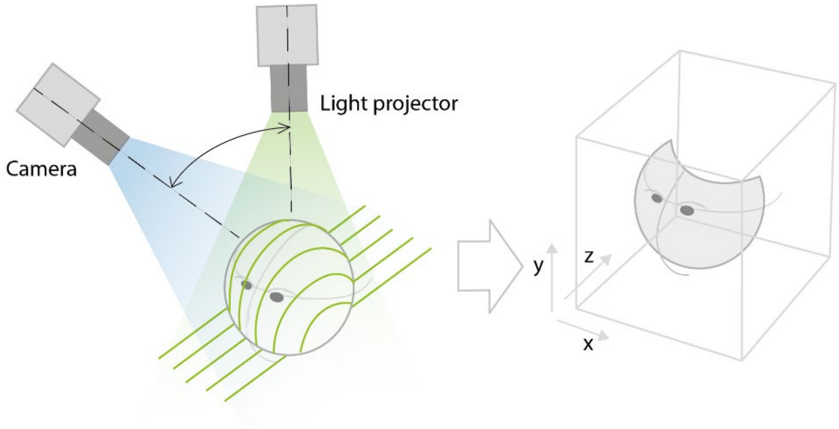

Fig 3. Structured light is deformed by the object. If one camera is used, the area that can be scanned is limited. Laser triangulation uses one projected laser line on the object, and to derive depth information the object has to be moved relative to the laser, for example on a turning table.

Handheld versus stationary	**Handheld** 3D scanning devices are portable devices and **kept in one or two hands during capture** (Fig 4 and 6). **Stationary** (Fig 5), including desktop scanners (Fig 7), have a **fixed position**, on the ground with tripods or as desktop device.	See Fig 4 and Fig 5 below
Capture method: point-and-shoot versus move-around-object, mosaic	**Point-and-shoot** means that **one capture** is taken, **that freezes the object in time** (Fig 6). Capture times can be short, allowing some movement of the object during capture. Point-and-shoot capture can be taken from one, narrow viewing angle, which is mostly the case with handheld devices pointed directly to the object, or wide viewing angle, for example 180 degrees or even 360 degrees. These wide viewing angles, are mostly stationary, fixed scanners using multiple cameras, for example from opposite directions, and the object must be placed in the 'viewing box' of the scanner. <br>Another capture method is **'move-around-object'** also referred to as **'mosaic' capture** (Fig 4): the **user moves the handheld 3D scanner slowly round the object** and the scanner builds a 3D surface in real-time, automatically stitching overlapping surfaces to one surface mesh of the object. This enables to obtain a 360 degrees scan of the object in one capture. Move-around-object captures can take several seconds, or even minutes, with the need of the object to stand still during capture.	See Fig 6 and Fig 7 below

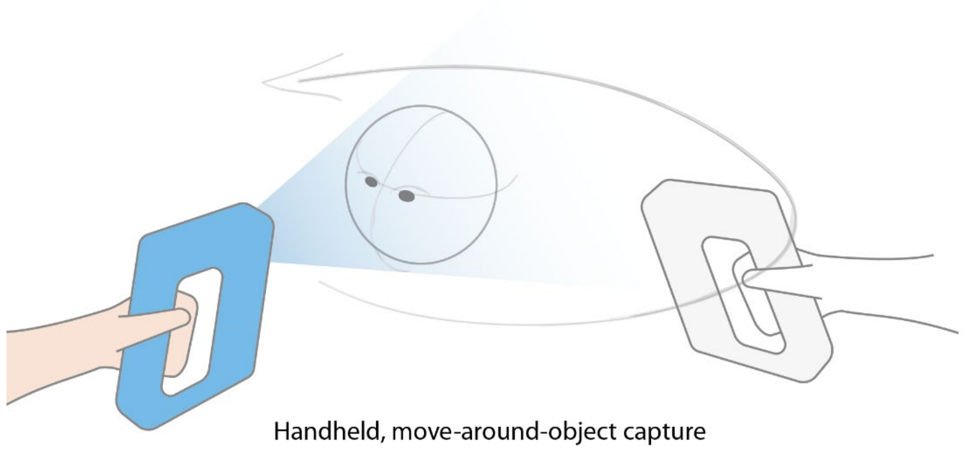

Fig 4. Handheld 3D Scanning. The device is held by hand and is moved around the object, or the object is moved in front of the scanner on a turning table. Because the capture-process takes time, the object should not move during capture, making it difficult to scan living subjects.

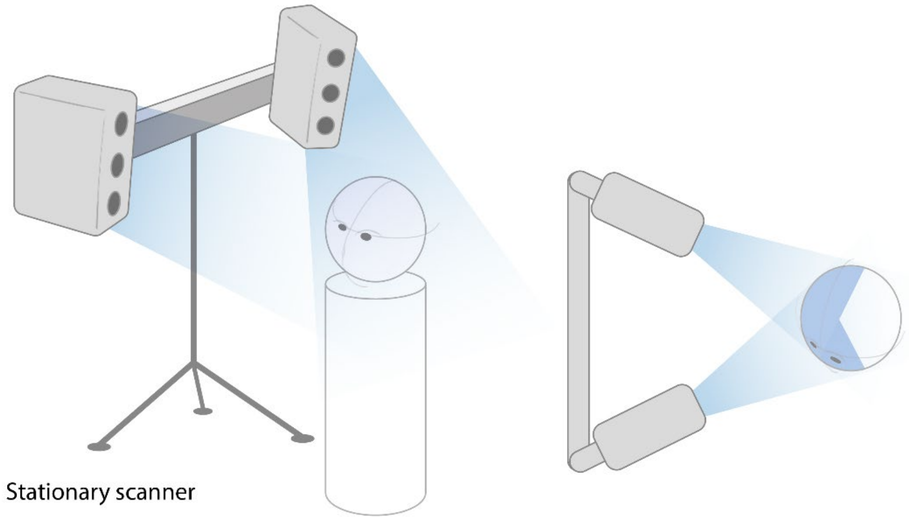

Fig 5. A stationary scanner can consist of several 3D cameras that capture the object from different viewing angles. To obtain a full 360 degrees scan, the cameras should completely surround the object, or the object is turned in front of the cameras on a turning table

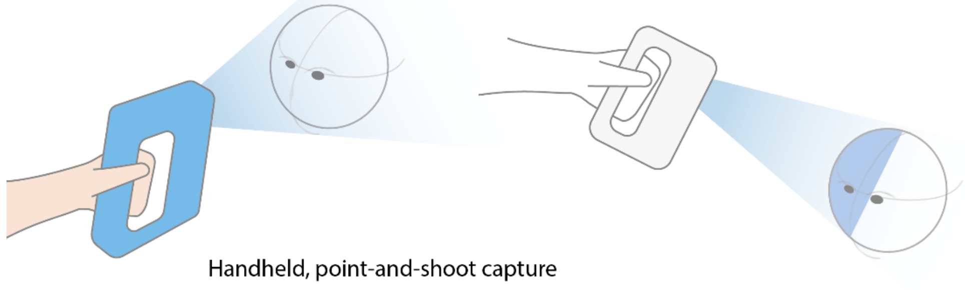

Fig 6. handheld scanning with point-and-shoot capture freezes a 3D-image of the object from one viewing angle. The short capture-time is convenient when scanning living subjects. However, this viewing angle limits the area of the 3D image. The short capture-times typically demands for additional (flash)light.

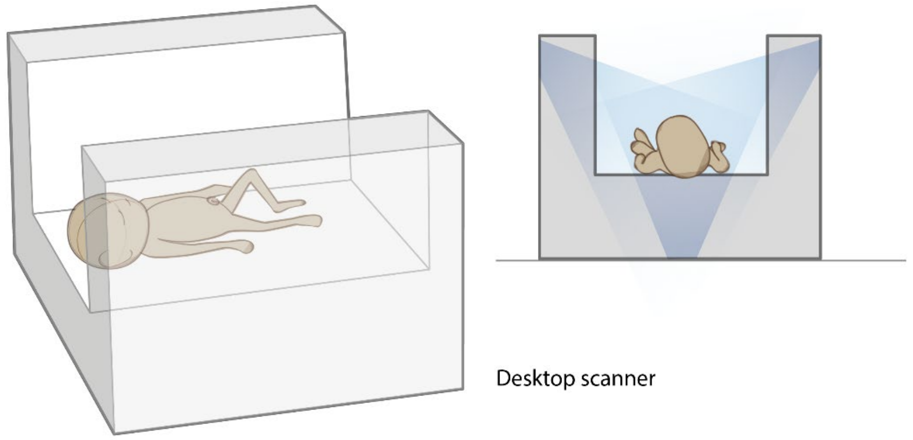

Fig 7. A desktop scanner has a dedicated area where the object should be placed. The scanner captures a 360 degrees 3D image with short point-and-shoot capture-times.

All illustrations: CC BY-NC RHJ van Gils 2022
