# Supplementary material for: Body size measuring techniques enabling stress-free growth monitoring of extreme preterm infants inside incubators: A systematic review
Source: PLoS One. 2022 Apr 22;17(4):e0267285. doi: 10.1371/journal.pone.0267285 (PMC9033282; doi:10.1371/journal.pone.0267285)
Supplement: S3 Appendix — (PDF) [file pone.0267285.s004.pdf]

Suitable techniques

VECTRA H1 handheld 3D scanner

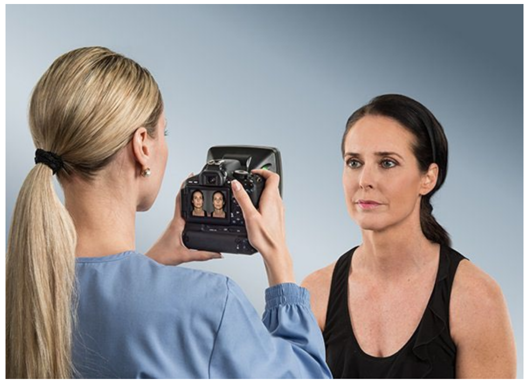

Image from:
https://www.canfieldsci.com/imaging-systems/vectra-h1-3d-imaging-system/

Stereoscopic vision system

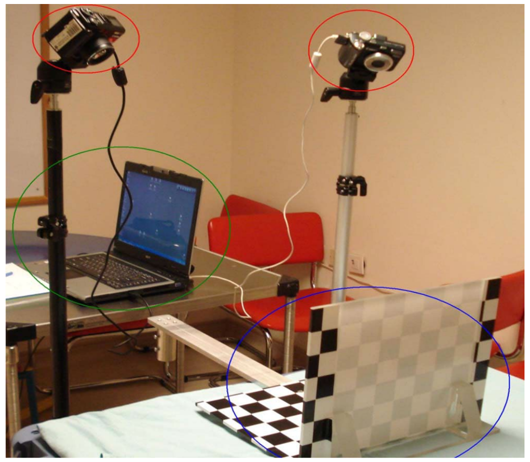

Stereoscopic Vision system with two cameras and calibration board (Image: Sokolover 2014), self-built device

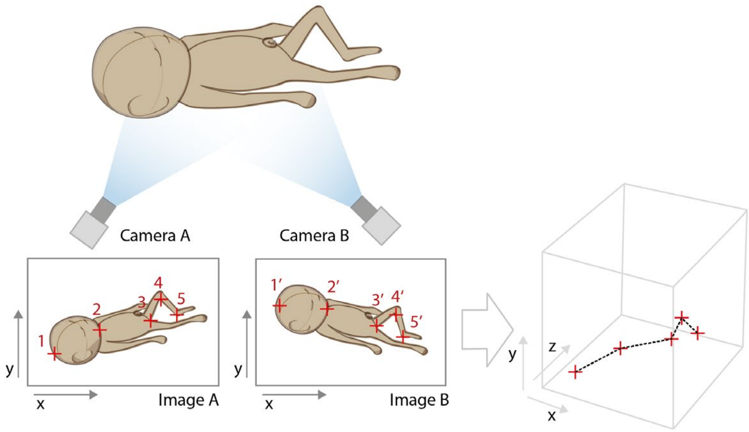

Illustration: CCBY RHJ van Gils 2022

Potentially suitable techniques

Scanify handheld 3D scanner

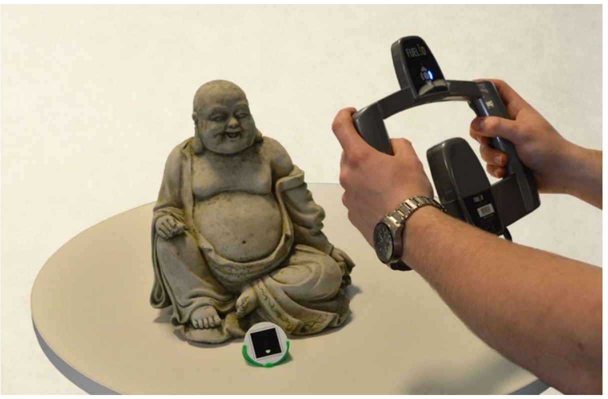

Image from:
https://www.dream3d.co.uk/product/fuel3d-scanify/

OMEGA handheld 3D scanner[a]

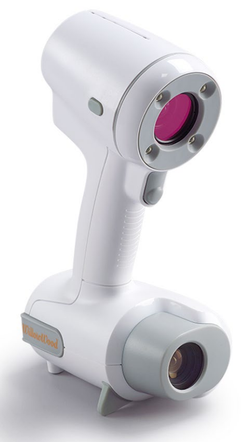

Image from:
https://www.willowwoodco.com/products-services/omega/hardware/omega-scanner-3d/

M4D handheld 3D scanner[a]

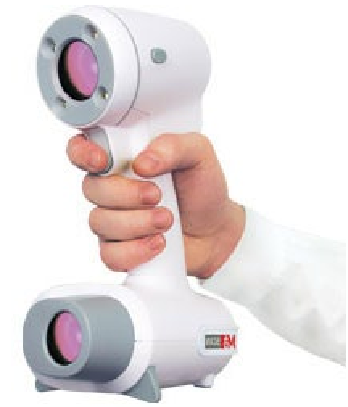

Image from:
http://rodin4d.com/en/Products/acquisition/m4d-scan

[a]The OMEGA and M4D seem technically identical.

Occipital Structure Sensor with iPad handheld 3D scanner with AutoAnthro software

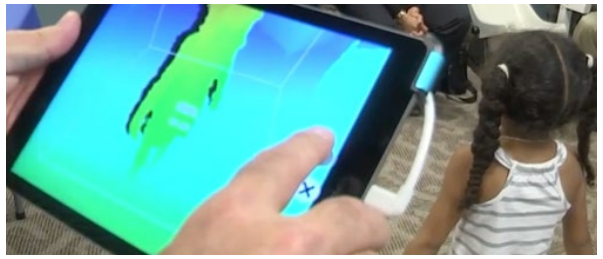

Image from:
AutoAnthro — Body Surface Translations

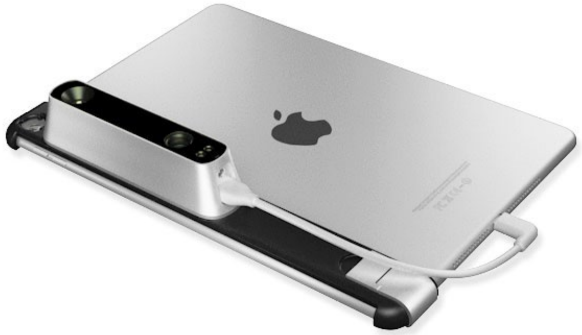

Image from:
https://structure.io/structure-sensor/getstarted

Smartphone handheld 3D scanning (via slow-motion video capture)

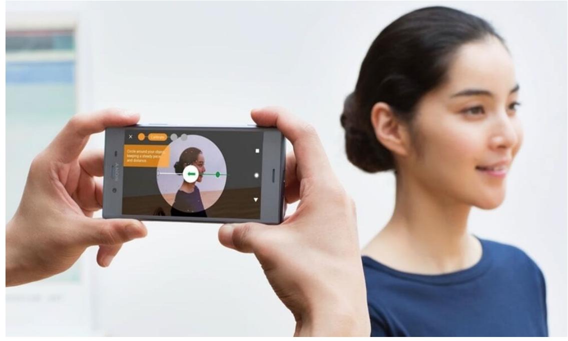

Image: Stock photo of Smartphone 3D scanning

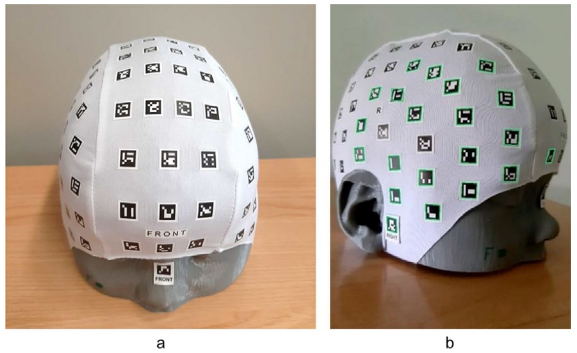

Coded cap, needed for 3D scanning with Smartphone slow-motion video (Image: Barbero-Garcia 2020)
