# Supplementary material for: Body size measuring techniques enabling stress-free growth monitoring of extreme preterm infants inside incubators: A systematic review
Source: PLoS One. 2022 Apr 22;17(4):e0267285. doi: 10.1371/journal.pone.0267285 (PMC9033282; doi:10.1371/journal.pone.0267285)
Supplement: S2 Data — (PDF) [file pone.0267285.s006.pdf]

S2 Data-table. Characteristics and Quality Assessment (QualSys)

First Author; year	Aim	Reported conclusion	Aim of study, categorized	Patients involved: Number, type, age	Type of body size measurement: Body Length (BL), Head Circumference (HC), Head Volume (HV) or Cranial Volume (CrV), etc.	Technology type (device model)	QualSys score Reviewer 1	QualSys score Reviewer 2
Andrews, E.T., 2019	To evaluate the feasibility and performance of 3D scanning as a 'non-touch' measuring technique for routine anthropometry.	High correlation between 3D data and standard measurement. 3D measures are not yet precise enough for routine clinical use. Refinement of technique/technology may translate into practical monitoring the growth of preterm infants with minimal handling and without interruption to developmental care	3D vs Manual	17 preterm infants born before 30 weeks gestation, mean 26 +0 (range 24 +0 29 +5); Corrected gestational age range at time of scan 24 +2, 41 +4 (mean 32 +1)	Head: Head circumference; Body length	3D Scanning: stereoscopic, photonic, handheld, point-and-shoot capture from one viewing point (SCANIFY)	0,85	0,90
Barbero-García, I., 2017	To develop a novel, low-cost, and minimally invasive methodology to correctly evaluate the cranial deformation using 3D imagery.	Smartphone-based photogrammetry is a low-cost, highly useful methodology to evaluate cranial deformation. This technique provides a much larger quantity of information than linear measurements with a similar accuracy as far as head marks exist. In addition, a new approach for the evaluation is pointed out: the comparison between the head 3D model and an ideal head, represented by a 3-axis ellipsoid.	3D vs Manual	5 children, one 6-month-old child; age of 4 others below one year.	Head: Cranial shape and size (perimeter)	3D Scanning: 3D photogrammetry, handheld (Smartphone), non-photonic: passive, slow motion video capture, 360 degrees scan by move-around-object capture (Samsung S7 Smartphone)	0,60	0,85
Barbero-García, I., 2020	In this paper, a novel, single photogrammetric smartphone-based solution for cranial deformation assessment is presented. A coded cap is placed on the infant's head and a guided smartphone app is used by the user to acquire the information, that is later processed on a server to obtain the 3D model. The smartphone app is designed to guide users with no knowledge of photogrammetry, computer vision or 3D modelling. The processing is all server-based and fully automatic.	This paper tackles the accuracy and repeatability analysis tested both for a single user (intra-user) and multiple non-expert user (inter-user) on 3D printed head models. The results allow us to confirm an accuracy below 1.5 mm, which makes the system suitable for clinical practice by medical staff. The obtained accuracy is higher to commonly used methodologies such as calliper and measuring tape, but it provides more complete information. Due to is low-cost, ease of use and reduced processing time, it is expected to be integrated as part of the clinical routine.	3D intra and inter-user reliability and accuracy (3D printed heads from CT/MRI reference scans)	None. Retrospective CT/MRI scans from 5 infants' heads were used to make 3D-printed reference heads., Author's comments: Postnatal	Head: Head shape	3D Scanning: 3D photogrammetry, handheld (Smartphone), non-photonic: passive, capture, 360 degrees scan by move-around-object capture	0,89	0,56

Brons, S., 2019	Aims of this study were: (1) to develop normative average 3D faces of healthy infants aged 3, 6, 9, and 12 months and (2) to describe normative average 3D facial growth data in infants aged 3 to 12 months.	The study describes the development of average faces at 3, 6, 9, and 12 months of age in healthy infants using 3D stereophotogrammetry. Normative data on the growth of the full face, nose, upper lip, chin, forehead and cheeks are presented. Such data can be used in future studies to identify the effectiveness of treatment of orofacial deformities such as orofacial clefts during the first year of life	3D shape analysis: (facial) growth	50 healthy new-borns recruited before the age of 3 months, Recruited before the age of 3 months. 3D images acquired at 3, 6, 9, and 12 months.	Head: Face dimensions	3D Scanning: stereophotogrammetric, stationary, 360 degrees scan in one capture. (3dMD Cranial System)	0,94	0,85
Burkhardt, W., 2019	1) To test the hypotheses that TBV – as determined by MRI – can be accurately estimated by CrV, measured by 3D-laser scanning.<br>2) Whether CrV can be also estimated by measuring HC in former preterm infants.<br>3) Assessment of other suitable technology than laser scanning for non-invasive measurement of CrV.<br>Method: a puppet head was measured, using different 3D-measurement principles: (i) Structured light projection system, (ii) The non-invasive laser-shape-digitizer, and (iii) Structure-from-motion (SFM) technique.	1) TBV can be estimated by CrV.<br>2) HC is poor predictor for CrV.<br>3) Techniques: besides laser shape digitizer, structured light projection and structure from motion are suitable in terms of accuracy. Robot arm laser light sheet scanner is not suitable due to long recording time. Time-of-flight camera not suitable due to poor accuracy.<br>Present and previous data suggest replacing measurement of HC by determination of CrV, since CrV reflects TBV more accurately than HC. 3D-scanning devices should be adapted to clinical requirements. Whereas the laser scanner provides a practical approach to obtain CrV in new-borns, it is more difficult to apply if the infant is dependent on intensive care interventions.	3D vs Manual, Correlation HC - CrV	1) MRI TBV and 3D scan CrV: 25 infants: median age of 19 month<br>2) For determine predictive value HC and CrV: in total 579 former Preterm infants were studied at 3, 6, 9 and 12 months of corrected age, representing a total of 579 laser scans and 539 manual measurements	Head: Total Brain Volume (TBV), Cranial volume (CrV) and Head circumference (HC)	3D scanning:<br>1) laser shape digitizer, stationary (desktop), photonic (laser light), 360 degrees scan in one capture (STARscanner)<br>2) structured light projection, stationary, photonic, point-and-shoot capture from one viewing angle (GOM ATOS Triple Scan II)<br>3) structure from motion, handheld, passive image capture, 360 capture by multiple images (Agisoft PhotoScan software)<br>4) laser light sheet scanner, robot arm, photonic (MicroScan 3D – RSI)	0,90	0,90
Conkle, J., 2019	Aim: to evaluate the efficiency, invasiveness, and user experience of the AutoAnthro System:<br>1) Time needed for a measurement compared to manual measurement;<br>2) User experiences, efficiency.<br>Author's comments: Other publications covered accuracy and reliability	Anthropometrists were not yet ready to completely abandon traditional, manual equipment for 3D scanners. For most children under 5 years of age, 3D imaging was an efficient and non-invasive way to capture anthropometric data. Revising the AutoAnthro system to address anthropometrists' concerns on capturing good quality scans of uncooperative children should help to facilitate widespread use of 3D imaging for child anthropometry in the health sector	3D vs Manual	36 children to calibrate system, 474 children in validation study, <5 years	Head: Head circumference;<br>Body length,<br>Arm circumference	3D Scanning: structured light (infrared) 3D scanner, photonic, handheld, move-around-object (mosaic) capture (Occipital Structure Sensor with AutoAnthro software)	0,80	0,65

de Jong, G., 2020	The goal of this study is to investigate if a deep learning algorithm is capable of correctly classifying the head shape of infants as either healthy controls, or as one of the following three craniosynostosis subtypes; scaphocephaly, trigonocephaly or anterior plagiocephaly.	This study shows that trained deep learning algorithms, based on 3D stereophotographs, can discriminate between craniosynostosis subtypes and healthy controls with high accuracy.	3D shape analysis (diagnose)	196 infants: 53 healthy controls mean infant; scaphocephaly 76 (10 earlier excluded); trigonocephaly 40 (4 earlier excluded); plagiocephaly 27 (3 earlier excluded), 3-6 months	Head: Head shape	3D Scanning: stereophotogrammetric, stationary, 360 degrees scan in one capture. (3dMD Cranial System)	1,00	0,64
Firmansyah, R., 2019	To make a tool aimed at incubators, that consists of three measurement parameters including weight, temperature, and head circumference. The tool can record the parameters automatically. The parameters automatically measure the infant's weight, temperature, and head circumference to determine the condition of the infant.	It is expected that this tool can help the paramedic work to act quickly because it can monitor the state of the infant online.	Ultrasonic vs ruler	No patients measured, N/A	Head: Head circumference	Ultrasonic, distance sensor (self-built)	0,06	0,07
Geil, M.D., 2008	Assessment of accuracy and reliability of measurement of the three-dimensional shape of a model of plagiocephalic infant head using a non-invasive laser shape digitalizer.	The results showed that the 3D-scanner may be used consistently by different practitioners on different days. Given that all three measurements showed sources of inconsistency with hand tools, it may be incorrect to label the hand tool measurement as a gold standard in this case. Although it is certainly the more common source of information, it is likely that, based on the consistency revealed in this investigation, the scanner results may be the more useful standard for comparison.	3D vs Manual	no infants involved, foam model head presenting a 10-month-old girl diagnosed with DP, foam model head of a 10-month-old female child with DP	Head: Head circumference, sellions landmarks at level 3, and cranial vault asymmetry index (CVAI)	3D Scanning, laser shape digitizer, stationary (desktop), photonic (laser light), 360 degrees scan in one capture (STARscanner)	0,90	0,45
Goto, L., 2019	To present up to date descriptive statistics of detailed measurements made of heads and faces of Dutch children.	This study shows that 3D photogrammetry offers an efficient way to scan babies and young children and facilitates this process because of its quick acquisition speed.	3D vs Manual	302 children, 0,5-7,9 years	Head: Head and face dimensions	3D Scanning, photogrammetry, stationary (3dMD Face System)	1,00	1,00

Ifflaender, S., 2013	1) To evaluate reproducibility of a 3D digital capture system in new-borns.<br>2) To compare manual and digital HC measurements in a neonatal cohort.<br>3) To determine correlation of HC and CrV and predictive value of HC.	3D CrV offers better growth monitoring data than 2D HC: Current practice of measuring frontal-occipational HC for describing head growth in preterm infants could be misleading since it does not present a three-dimensional approach.<br>The 3D laser-scanning device represents a new and promising method to provide reproducible data of cranial volume and head circumference	3D vs Manual, Correlation HC - CrV	282 infants, preterm and term infants, at birth: median 34 _+2 (IQR 32 +0 - 36 +0); at measurement median 45 _+6 (min 31_+ 2, max 46 _+6)	Head: Head circumference and Head volume (CrV)	3D Scanning: laser shape digitizer, stationary (desktop), photonic (laser light), 360 degrees scan in one capture (STARscanner)	1,00	0,85
Linz, C., 2014	To evaluate a standardized operative procedure to correct premature sagittal craniosynostosis using non-invasive 3D stereophotogrammetry before and after surgical correction.	3D stereophotogrammetry is a reliable and a valuable tool with no side effects: "This study demonstrates the superiority of 3D stereophotogrammetry compared to other analytic tools."<br>Procedure showed good postoperative results with decreased length and increased width and an improved cranial index.	3D shape analysis: operative result	20 children (3 girls and 17 boys), mean age of 7.3 months	Head: Head volume (Cranial volume), Head circumference, width, length, max width and length (Cranial index), total cranial volumes of Q1 - Q4	3D Scanning: stereophotogrammetric, stationary, 360 degrees scan in one capture. (3dMD Cranial System)	1,00	0,60
Martini, M., 2018	To evaluate the validity of maximal head circumference to cranial volume in the first year of life using a new method which includes ear-to-ear over the head distance and maximal cranial length measurement.	The results demonstrate that for a distinct improvement in the evaluation of a physiological cranial volume development, the additional measurement of the ear-to ear distance using a measuring tape is expedient, and, especially for cases with pathological skull changes, such as craniosynostosis, ought to be conducted.	correlation CrV - HC vs correlation HC & ear-to-ear & 'head length', 3D as reference	44 healthy Caucasian infants (29 male, 15 female), 4 and 12 months	Head: Head circumference; ear-to-ear over the head distance; maximal cranial length measurement; cranial volume	3D Scanning: structured light, photonic, stationary*? (3D-Shape*)<br>* device model not reported	1,00	1,00
Meyer-Marcotty, P., 2014	To generate a 3D longitudinal database of cranial growth using a non-invasive approach.	The 3D data generated in this study for the entire heads of healthy infants could be used for diagnostic evaluation and to analyse the growth of the cranium from 6 to 12 months of age. Moreover, these normative data are clinically helpful in the correction of cranial asymmetries induced by craniosynostosis or positional plagiocephaly.<br>Good reproducibility for each 3D parameter	3D shape analysis: growth	52 Caucasian infants (27 females and 25 males), 6 to 12 months of age, mean (T1) was 6.3±0.5 months, mean age (T2) was 11.5±0.6 months	Head: CrV	3D Scanning: stereophotogrammetric, stationary, 360 degrees scan in one capture. (3dMD Cranial System)	1,00	0,90

Meyer-Marcotty, P., 2018	Longitudinal three-dimensional (3D) data investigating the physiological growth of the infant skull.	This prospective study is the first longitudinal 3D analysis to examine the physiological growth dynamics of infants' heads within the first months of life. Understanding patterns of skull growth in all three dimensions is important for gaining further insights into physiological and pathophysiological skull development.	3D shape analysis: growth	40 Caucasian infants (19 male, 21 female), 4 months	Head: CrV	3D Scanning: stereophotogrammetric, stationary, 360 degrees scan in one capture. (3dMD Cranial System)	1,00	0,85
Nahles, S., 2018	To compare conventional anthropometry and laser scanning, two different measurement methods, as diagnostic instruments for plagiocephaly. The present study also tests the measurement time of both methods and whether one method is easier on the patient than the other.	In comparison with the conventional anthropometric method, measurements made with a 3D laser scanner yield inconsistent results. Moreover, the current state of technology of 3D cephalometry has no advantages compared with the conventional anthropometric method. Disadvantages worth mentioning appear to be the higher technical outlay and the considerable acquisition, service, and maintenance costs.	3D vs Manual	44 children (21 girls, 23 boys), diagnosed with plagiocephaly, mean age 8.8 months, youngest 4.6 months and oldest 14.2 months	Head: Head circumference, Head length, Head width, Head diagonals	3D Scanning: handheld, structured light, photonic, can make 360 degrees scan with mosaic move-around-object capture (OMEGA)	1,00	0,90
Ritschl, L.M., 2018	To evaluate the feasibility and accuracy of a low-budget portable system for 3D image acquisition with special regard to the gracile nasal region in neonates. Furthermore, the study aimed to establish a 3D data set of the first 180 days post-partum.	The analysed portable 3D stereophotogrammetry system is a feasible methodology with good accuracy, even in new-born. A description of the growth as well as the establishment of a 3D data set was performed. 3D data might reduce the need for impressions and facilitate the communications with parents and the interdisciplinary team.	3D vs 3D reference scan of plaster impression model	33 neonates (16 males and 17 females), full-term new-borns, 0-6 months	Head: Face dimensions, perinasal area	3D Scanning: photogrammetry, photonic, handheld, point-and-shoot capture gives 3D scan from one viewing angle. (SCANIFY)	1,00	0,85
Santander, P., 2019	To evaluate a contactless 3D imaging system to assess head shape and volume in preterm infants:<br>1) to validate a stereophotogrammetric por- table camera, 2) to evaluate the feasibility of the imaging system in clinical routine and 3) to compare manual versus digital HC measurement.	The portable camera system allowed fast and contactless 3D image capture of a preterm infant's head without any risk or interference with neonatal care. Together with a new software, this technique would allow more precise evaluation of head growth even in very preterm infants and thereby may improve their care and long-term outcome.<br>The portable camera had a higher detailed accuracy than the stationary camera. The only current drawback is the long processing time for image reconstruction.	3D vs Manual	six preterm infants, five female, mean gestational age 33.2 ± 1.5 weeks; 3D reconstructions before a corrected gestational age of 37 weeks (mean 35.3 ± 1.5 weeks)	Head: HC, Head shape, CrV	3D Scanning: Stereophotogrammetry with added flash, handheld, point-and-shoot. 10 separate captures needed to assemble a 360 degrees scan (VECTRA H1)	0,95	1,00

Schaaf, H., 2010	Investigation of the accuracy of 3D photogrammetry in cranial deformities in terms of the repeatability of measurements and their accuracy compared with anthropometric measurements using callipers under clinical conditions.	3D photogrammetry is potentially a reliable tool for treatment planning and follow-up of abnormal head shapes in infancy	3D vs manual; 3D Shape analysis: treatment planning	100 children with non-synostotic cranial deformities. Plagiocephaly n=52, brachycephaly n=8, combined plagiocephaly and brachycephaly n=40, 4-20 months. Median age at the initial examination: plagiocephaly group 31.5 wks; brachycephaly group 31.5 wks ; and 27.4 wks for the combined group	Head: Cranial shape/volume/size, cranial vault asymmetry index (CVAI)	3D Scanning: photogrammetry, stationary, 360 degrees scan in one capture. (3dMD Cranial System)	1,00	0,85
Schloesser, R.L., 2011	To determine Body surface area (BSA) in healthy term and near-term neonates by 3D scanning, as a suitable alternative to calculation with mathematical formulae.	3D scanning is an accurate and practical method to estimate BSA in new-borns. Individual and repeated measurements from day to day are possible. Further studies are warranted in preterm and sick neonates.	3D vs calculation	209 term or near-term infants, healthy<br>53 analysed, mean gestational age was 39 weeks (range 35–42)	Body surface area	3D Scanning: stationary desktop setting, structured light, photonic. One scan covers 180 degrees by use of two mirrors (3D-Shape custom-built)	1,00	0,80
Sokolover, N., 2014	To improve infant length measurement by development of a novel, accurate, precise and practical measurement technique.	Precision of the new technique was demonstrated by a technical error of measurement of 2.57 mm. Conclusions: The stereoscopic system is accurate, reliable, easy to use, and involves less handling and discomfort to the new-borns. It has the potential to measure premature infants or sick neonates through incubators.	3D Stereoscopic vs manual	20 full-term infants and 34 preterm infants (25 females and 29 males), Full term: postmenstrual age between 34 and 49 weeks.<br>preterm infants (gestational age range, 25–36 weeks)	Body length	Stereoscopic Vision, stationary, non-photonic, passive still photography (self-built)	1,00	0,91
Tenhagen, M., 2016	To assess the utility of 3D handheld scanning photography in a cohort of patients who underwent spring-assisted correction surgery for scaphocephaly.	No significant differences were observed in the CI between 3D and x-ray. Three-dimensional handheld scanning followed by SSM proved to be an efficacious and practical method to evaluate 3D shape outcomes after spring-assisted cranioplasty in individual patients and the population.	3D vs X-ray	9 children (1 female), 4-7 months	Head: Head circumference, sagittal length, coronal width, cranial volume	3D scanning: handheld structured light 3D scanner, photonic, 360 scan with mosaic move-around-object capture (M4D Scan)	1,00	0,73

Tu, L.Y., 2020	Background: Current methods to analyse three-dimensional photography do not quantify intracranial volume, an important metric of development. This study presents the first non-invasive, radiation-free, accurate, and reproducible method to quantify intracranial volume from three-dimensional photography.	Three-dimensional photography with image analysis provides measurement of intracranial volume with clinically acceptable accuracy, thus offering a non-invasive, precise, and reproducible method to evaluate normal and abnormal brain development in young children	3D intracranial volume prediction model vs CT-scans	575 Children retrospective CT;<br>30 children retrospective CT and 3D photography,<br>CT scans: average age, 5 ± 5 years; range, 0 to 16 years;<br>CT scans and 3D photography: average age, 1 ± 3 years; range, 0 to 9 years	Head: Intracranial volume, Head volume	3D Scanning: stereophotogrammetric, stationary, 360 degrees scan in one capture. (3dMD Head System)	1,00	0,85
Vermeulen, M.J., 2021	A new reliable proxy for brain volume is cranial volume, which can be measured routinely by 3-D laser scanning. The aim of this study was to develop reference charts for normal cranial volume in new-born infants at different gestational ages starting from late preterm for both sexes.	Earlier studies showed that 3-D laser scanning is an easy and accurate method to measure neonatal cranial volume. We now provide reference charts and tables to be used for cranial volume monitoring during routine neonatal care. These reference values may be used to support development of new clinical interventions, such as personalized nutritional care, to improve brain growth and later outcome after preterm birth.	3D shape analysis (growth)	1703 infants, 34 to 42 weeks GA	Head: Cranial volume	3D Scanning, laser shape digitizer, stationary (desktop), photonic (laser light), 360 degrees scan in one capture (STARscanner)	1,00	1,00
Wang, J.C., 2000	To assess the accuracy of a metric photographic height estimation method.	Without any high technology equipment, this simple approach can be readily applied to obtain satisfactory estimates for epidemiological studies. Accuracy depends on accurate measure/ estimate of reference object. In this study accuracy of 1,5 cm was achieved. The mean differences between the measured and estimated heights were 1.4 cm and 1.5 cm respectively, and both were not statistically significant. Time between measured height and the photo was maximum two months. These two months can give an undesired difference between estimated height on the photo and real measured height because the child can grow significantly during two months period.	2D linear metric vs manual	23 (photographs of) Children, 0-12 years	Body length	2D Linear metric, measure from existing photographs (no device used)	0,90	0,85

Weinberg, S.M., 2006	To compare anthropometric measurements obtained by way of two different digital 3D photogrammetry systems (Genex and 3dMD) as well as direct anthropometry (calliper) and to evaluate intra-observer precision across these three methods. Method: On a sample of 18 mannequin heads, 12 linear distances were measured twice by each method.	Results indicate that overall mean differences across these three methods were small enough to be of little practical importance. In terms of intra-observer precision, all methods fared equally well	3D vs 3D vs manual	No patients: 18 mannequin heads, each with 17 standard surface landmarks prelabelled in permanent ink, adult mannequin heads??	Head: Face dimensions	3D Scanning:<br>1) photogrammetry, stationary, photonic, structured light, capture from one viewing point (Genex)<br>2) photonic unstructured light, stationary, 180 degrees (ear-to-ear) capture (3dMD Face System)	0,75	0,80
