# Supplementary material for: Body size measuring techniques enabling stress-free growth monitoring of extreme preterm infants inside incubators: A systematic review
Source: PLoS One. 2022 Apr 22;17(4):e0267285. doi: 10.1371/journal.pone.0267285 (PMC9033282; doi:10.1371/journal.pone.0267285)
Supplement: S3 Data — (PDF) [file pone.0267285.s007.pdf]

S3 Data-table. Body size measurement technology type

First Author; year	Type of body size measurement: Body Length (BL), Head Circumference (HC), Head Volume (HV) or Cranial Volume (CrV), etc.	Technology type (device model)	Technology description and (manufacturer) specifications	(Reported) accuracy
Andrews, E.T., 2019	Head: Head circumference; Body length	3D Scanning: stereoscopic, photonic, handheld, point-and-shoot capture from one viewing point (SCANIFY)	SCANIFY (FUEL 3D), a handheld point-and-shoot 3D scanner using a stereo optical camera set-up, was used to carry out 3D scans. HC and body length derived via digital measuring tool (SCANIFY software), on the 3D image 1D distances can be derived by manually selecting a contour by point selection.<br>Author's comment: The 3D pictures lack the underside of the patient relative to the scanner. Length was measured using the SCANIFY 3D (software) measuring tool following a series of bony landmarks that attempted to allow for flexed legs. This was in an effort not to disturb the infants but take a proxy measure in the position they lay. The head circumference was measured looking "top down" which was able to be captured relatively easily looking from the head end of the patient toward the feet. As both of these images were able to be viewed and measured on a 3D platform it was possible to attempt to gain proxy measurements for HC and length although as the reviewers comment there was discrepancy between image captured measured length and manually measured length.	Reported accuracy:<br>Mean Difference manual - scan measure (95%CI):<br>HC 1.8 mm (0.6 to 4.2); length 2.7 mm (0.3-5.4)<br>Mean residual difference between manual and scan measure (95%CI) HC 7.7 mm (6.1-9.3) Length 10.1 mm (9.4-12.2).
Barbero-García, I., 2017	Head: Cranial shape and size (perimeter)	3D Scanning: 3D photogrammetry, handheld (Smartphone), non-photonic: passive, slow motion video capture, 360 degrees scan by move-around-object capture (Samsung S7 Smartphone)	Handheld, Smartphone based photogrammetry. Cap with markers (stickers) placed on head, to allow scaling of 3D model. slow-motion video capture of the head with Samsung S7 smartphone. Separate 2D images were extracted from this slow-motion video, and masked (only head visible, rest darkened). To obtain an accurate 3D model, the images must have a good geometric distribution and cover the whole head.<br>3D model creation with Agisoft Photoscan software (Agisoft LLC, St. Petersburg, Russia). 3D Visualization (distances to ellipsoid reference) with CloudCompare 2.7.0.	Accuracy: a 3D-mesh was created from the (photometric) point cloud, the mesh was scaled with the use of reference marks placed on the head, that resulted in an accuracy of better than 1mm for 'three distances measured'.
Barbero-García, I., 2020	Head: Head shape	3D Scanning: 3D photogrammetry, handheld (Smartphone), non-photonic: passive, capture, 360 degrees scan by move-around-object capture	The PhotoMeDAS (Photogrammetric Medical Deformation Assessment Solutions) patent-pending tool is composed of coded cap, coded stickers, mobile app and processing software. The mobile application is used together with the cap and stickers to carry out the data acquisition. The software, located in a server, processes the data and creates a 3D model. It also obtains some head shape information and deformation parameters, which are available to the user through a webpage service;<br>Capture: More than 200 images covering the totality of the model are required to assure a good orientation (Barbero-García et al., 2018). Once the required number of images is registered and the whole head is correctly covered, the app will automatically send the data for processing.<br>Postprocessing: combination of open-source software and ad-hoc developed software: The software MicMac (Pierrot Deseilligny and Clery, 2012), and, specifically, the tools Tapas and AperiCloud, are used to obtain the 3D point cloud. MicMac tool Tapas: camera calibration. Once the frames are oriented, the point cloud is then obtained using AperiCloud. The resulting point	The mean (intra/inter-user) accuracy is 0.5 ± 0.4 mm, and the (intra/inter-user) repeatability differences are 0.3 ± 0.3 mm

			cloud is scaled using the markers size and the point normals are computed automatically using MeshLab 1.3.3	
Brons, S., 2019	Head: Face dimensions	3D Scanning: stereophotogrammetric, stationary, 360 degrees scan in one capture. (3dMD Cranial System)	3dMDcranial system. 3D facial images were constructed from the 2D images acquired by 15 digital cameras using the 3dMDpatient 4.0 software. The selected 3D images were first exported from the 3dMDpatient 4.0 software as wave front object (.obj) files with texture. Following this, the 3D images were imported into Maxilim version 2.3.0.3 (Nobel-Biocare, Mechelen, Belgium). The children's reference frame described by Brons et al. was used to align all 3D images in the correct position and orientation. Using the Meshmixer software (Autodesk), remeshing of the 3D images was performed to obtain a uniform mesh pattern with polygon edges of1.5 mm. MATLAB (MathWorks, MA, USA) was then used to automatically annotate the left and right pupils, the pronasale, and the left and right exostomion on the aligned 3D images. The landmarks were indicated on the 2D texture files automatically with a cascaded convolutional network described by Zhang et al. and transferred to the 3D images	Not reported. "High quality 3D images were acquired". Measured growth is presented in tenths of millimetres.
Burkhardt, W., 2019	Head: Total Brain Volume (TBV), Cranial volume (CrV) and head circumference (HC)	3D scanning:<br>1) laser shape digitizer, stationary (desktop), photonic (laser light), 360 degrees scan in one capture (STARscanner)<br>2) structured light projection, stationary, photonic, point-and-shoot capture from one viewing angle (GOM ATOS Triple Scan II)<br>3) structure from motion, handheld, passive image capture, 360 capture by multiple images (Agisoft PhotoScan software)<br>4) laser light sheet scanner, robot arm, photonic (MicroScan 3D – RSI)	TBV by MRI scanner.<br>Head measurements (CrV) with 3D scan technologies:<br>1) Non-invasive laser shape digitizer (STARscanner Vorum research Corp., Vancouver, BC);<br>2) Structured light projection system (GOM ATOS Triple Scan II, GOM GmbH Germany), consisting of two cameras and a projector;<br>3) Structure-from-motion (SFM) techniques generate a 3D point cloud based on multiple images of an object taken from different camera positions. The automatic generation of 3D-point clouds of the objects was done with the software package Agisoft PhotoScan (now: Agisoft Metashape) (St. Petersburg, Russia).<br>Failed technologies:<br>4) A robot arm laser light sheet scanner (MicroScan 3D – RSI GmbH), turned out not to be suitable for the acquisition of new-borns head due to its long recording time;<br>5) Time-of-Flight depth camera (PMD CamCube 2.0): "the point cloud data recorded showed insufficient accuracies."	1) STARscanner: "radial coordinate differences were mainly in an interval of±0.3mm. the overall standard deviation improved from 0.46mm to 0.38 mm" (after removal of 'obviously erroneous areas' due to resolution of device) "These values are in accordance with accuracy specification of the manufacturer (0.5 mm)"<br>2) Structured light projection system: Manufacturer claimed accuracy 0,1mm: These 3D scan were used as the reference 3D images to assess to accuracy of the other assessed scan technologies.<br>3) Structure-from-motion: "standard deviations of the difference were ± 0.57mm and thus slightly higher than for the non-invasive laser shape digitizer. After an interactive elimination of obviously erroneous areas, the standard deviation dropped to±0.27 mm"
Conkle, J., 2019	Head: Head circumference;<br>Body length,<br>Arm circumference	3D Scanning: structured light (infrared) 3D scanner, photonic, handheld, move-around-object (mosaic) capture (Occipital Structure Sensor with AutoAnthro software)	AutoAnthro (BST, Atlanta, GA), a custom software developed by Body Surface Translations for capturing and processing scans using the tablet-based Structure Sensor (Occipital, San Francisco, USA), that is attached to an iPad tablet. The structure sensor uses the RGB camera of the tablet.	Accuracy: not reported in detail in this study but a previous publication (Conkle 2018) focused on technique validation regarding accuracy and reliability. Author's comments: reliability was okay, but more work was needed to ensure accuracy. Also, there was a follow up study on accuracy and reliability by Emory/Gates (pending publication).
de Jong, G., 2020	Head: Head shape	3D Scanning: stereophotogrammetric, stationary, 360 degrees	3D photography: 3dMDCranial 3DMD, Atlanta, USA;<br>Automatic diagnose with artificial intelligence: We used a deep learning network with a categorical outcome, which classified the data of each subject as one of the following: healthy, scaphocephaly, trigonocephaly, anterior plagiocephaly. The used network was a conventional,	3D photography: not reported.<br>Deep learning network: Out of 196 patients and healthy controls, 195 subjects (99.5%) were correctly classified; precision 98.1% specificity 99.2%

		scan in one capture. (3dMD Cranial System)	feed-forward neural network holding respectively 192, 128, 64 and 32 nodes within the hidden layers. The software used for the deep learning network creation was Keras42 with the Tensorflow43 backend. The software used for statistical analysis of the deep learning results and pre-processing of the data was Scikit-learn.	
Firmansyah, R., 2019	Head: Head circumference	Ultrasonic, distance sensor (self-built)	The tool consists of a NodeMCU ESP8266 module using two ultrasonic sensors to determine the circumference of the infant's head, the weight sensor using load cell, and the temperature sensor using an SHT31 temperature sensor. NodeMCU ESP8266 is used to send the data from sensors to a laptop or smartphone through internet Wi-Fi network. The tool is operated through IoT (internet of things) which can facilitate paramedic to monitor the infant's situation wherever and whenever through internet network that can be accessed via web or Android.	From the five measured data and comparison data three data differ 1 cm, two data zero cm: an inaccuracy of 1 cm.
Geil, M.D., 2008	Head: Head circumference, sellions landmarks at level 3, and cranial vault asymmetry index (CVAI)	3D Scanning, laser shape digitizer, stationary (desktop), photonic (laser light), 360 degrees scan in one capture (STARscanner)	STARscanner Laser Data Acquisition System (Orthomerica)	STARscanner Manufacturer specifications claim an accuracy of 0,5 mm
Goto, L., 2019	Head: Head and face dimensions	3D Scanning, photogrammetry, stationary (3dMD Face System)	3dMD Face system scanner. The four 3D images that were captured were combined in Artec Studio 9 software (Artec group, Luxembourg) to obtain a complete 3D image of the participant. Remaining holes in the image were repaired in Geomagic Studio 2013 software (3D Systems, Rock Hill, SC, USA). All images were aligned with MATLAB™ 2015a software. The 3D images were manually landmarked using 3dMDvultus 2.1 software	3dMD Face sys. accuracy (geometric accuracy of 0.2 root mean square)
Ifflaender, S., 2013	Head: Head circumference and Head volume (CrV)	3D Scanning: laser shape digitizer, stationary (desktop), photonic (laser light), 360 degrees scan in one capture (STARscanner)	A non-invasive laser shape digitizer (STARscanner, Orthomerica, Orlando, FL, USA) was used to acquire digital HC and volume data. The device captures a three-dimensional infant head shape using four Class-I eye-safe lasers that create circumferential light beams around the surface of the cranium. Eight cameras reconstruct the surface.<br>To acquire HC and CrV from the 3D scan, 3D postprocessing is needed with two software applications:<br>1) to place landmarks, three anatomical landmarks are manually marked in the computer on the 3D scan (YETI Shape Builder software, Vorum)<br>2) to divide in cross sections to define resp. calculate the cranium volume and HC. (Cranial Comparison Utility, Vorum Research Corporation, Vancouver, Canada).	STARscanner Manufacturer specifications claim an accuracy of 0,5 mm
Linz, C., 2014	Head: Head volume (Cranial volume), Head circumference, width, length, max width and length (Cranial index), total cranial volumes of Q1 - Q4	3D Scanning: stereophotogrammetric, stationary, 360 degrees scan in one capture. (3dMD Cranial System)	The entire head was recorded circularly and contact-free using a specially developed stereophotogrammetric scanner for synchronized cameras (3dMD, Atlanta, GA, USA). 360°-data acquisition is achieved in a standardized protocol in only one scan with a recording time of 1.5 ms. After triangulation and editing, the data sets were converted to a common 3D data format (.stl) and analysed with special 3D software (Analytics 3.0, Cranioform Alpnach, Switzerland).	(Absolute) instrument accuracy not reported. High, proven accuracy of 3dMD cranial system scanner is assumed in this study. (Refers to Schaaf 2010)
Martini, M., 2018	Head: Head circumference; ear-to-ear over the head distance; maximal cranial length measurement; cranial volume	3D Scanning: structured light, photonic, stationary*? (3D-Shape*)<br>* device model not reported	3D optical image scans of the cranium and facial surface, with the help of an optical 3D sensor (3D–Shape®, Erlangen, Deutschland). These data were triangulated and fused using Software Slim3D (3D–Shape, Erlangen, Deutschland).<br>After converting to a STL- format, cephalometric analysis of the data followed with the help of Software Onyx Ceph (Image Instruments GmbH, Chemnitz, Deutschland).	(Absolute) instrument accuracy not reported. High, proven accuracy of 3D-Shape scanner is assumed in this study.

Meyer-Marcotty, P., 2014	Head: CrV	3D Scanning: stereophotogrammetric, stationary, 360 degrees scan in one capture. (3dMD Cranial System)	Data on the heads were generated with a 360 scanner (3dMD®cranial System, Atlanta, GA, USA) based on non-invasive stereophotogrammetric imaging. After triangulation and editing, the scans were converted to a common 3D data format (.stl) and analysed with Cranioform Analytics 4.0 3D software (Cranioform®, Alpnach, Switzerland).	(Absolute) instrument accuracy not reported. High, proven accuracy of 3dMD cranial system scanner is assumed in this study.
Meyer-Marcotty, P., 2018	Head: CrV	3D Scanning: stereophotogrammetric, stationary, 360 degrees scan in one capture. (3dMD Cranial System)	Data acquisition: Data on the heads were generated with a 360 scanner (3dMD®cranial System, Atlanta, GA, USA)<br>Data analysis: After triangulation and editing, datasets were converted into a common 3D-format and analysed using the Cranioform Analytics 4.0 3D-software (Cranioform®, Alpnach, Switzerland).	(Absolute) instrument accuracy not reported. High, proven accuracy of 3dMD cranial system scanner is assumed in this study.
Nahles, S., 2018	Head: Head circumference, Head length, Head width, Head diagonals	3D Scanning: handheld, structured light, photonic, can make 360 degrees scan with mosaic move-around-object capture (OMEGA)	3D hand laser scanner OMEGA® (Ohio, USA) (frequency of 10-500 KHz) with the integrated software OMEGA® Tracer®, WillowWood (Ohio, USA). The scanner is moved around the object at a distance of 30-40 cm.<br>Parameters were measured from the 3Dimage using "a virtual measuring tool". It is not reported how and how much time needed to derive the metric parameters from the 3D image.	According to the manufacturer, the instrument is accurate to within 0.5 mm over the entire surface.
Ritschl, L.M., 2018	Head: Face dimensions, perinasal area	3D Scanning: photogrammetry, photonic, handheld, point-and-shoot capture gives 3D scan from one viewing angle. (SCANIFY)	Portable low-budget 3D stereophotogrammetry system FUEL3D SCANIFY. The 3D scans were further reprocessed by the corresponding software FUEL3D Studio 2.2 Professional, resulting in STL and Polygon File Format (.PLY) files. For automatic dimensioning a target reference is placed near the object.<br>3dMDvultus software for analysing landmarks on 3D images.<br>Scanner used for reference 3D scans of plaster moulds: dental Laser-Scanner (3Shape D700, 3Shape® A/S, Denmark), accuracy of 20 µm.<br>Geomagic software to compare 3D images of Scanify handheld scanner with accurate 3D reference scans of plaster models: "Root Mean Squared error (RMS error) was 0.72 ± 0.22 mm between the superimposed surfaces."	SCANIFY accuracy: "The focus is precalibrated and captures 3D data at a diagonal of circa 40 cm with a resolution of up to 350 µm resulting in up to 375,000 polygons per scan"<br>Scanner used for reference 3D scans of plaster moulds: dental Laser-Scanner (3Shape D700, 3Shape® A/S, Denmark), accuracy of 20 µm.<br>Geomagic software to compare 3D images of Scanify handheld scanner with accurate 3D reference scans of plaster models: "Root Mean Squared error (RMS error) was 0.72 ± 0.22 mm between the superimposed surfaces."
Santander, P., 2019	Head: HC, Head shape, CrV	3D Scanning: Stereophotogrammetry with added flash, handheld, point-and-shoot. 10 separate captures needed to assemble a 360 degrees scan (VECTRA H1)	Handheld 3D camera: Vectra H1 camera (Canfield Scientific, New Jersey, USA), a portable 3D imaging system based on stereophotogrammetry. The system is attached to a Canon SLR casing (Canon, Krefeld, Germany) with a special flash device. During each single shot, the camera records two images from different constant angles with a field of 165mm width, 270mm height and 100mm depth. In total, ten separate images are needed for an entire head and face 3D reconstruction.<br>The image processing: 1) separation of the study object from the background using the VECTRA Analysis Module (VAM) version 6.2.3 software (Canfield Scientific, New Jersey, USA). Large vertices caused by shadows as well as invalid vertices were removed manually. 2) to assemble two images using the MeshLab version 2016.12 software (Visual Computing Lab, ISTI – CNR, Pisa, Italy). Landmarks on the nylon caps facilitated (manually) find the overlap in two surfaces. 3) compare the complete 3D image with a reference image with 3-matic Research version 13.0 Software (Materialise Leuven, Belgium).<br>Measurements: HC was digitally derived from the 3D images using Cranioform software version 4.0 (Cranioform, Alpenach Switzerland).	Accuracy: When comparing the automatic reconstruction of the M5 stationary system and the manual reconstruction of the H1 portable camera mean differences using part comparison (point to point evaluation) differences of 0.21mm ± 0.03mm were recorded. "When comparing manual to digital HC measurement, the results obtained by 3D stereophotogrammetric technique were superior with <1mm deviation".

Schaaf, H., 2010	Head: Cranial shape/volume/size, cranial vault asymmetry index (CVAI)	3D Scanning: photogrammetry, stationary, 360 degrees scan in one capture. (3dMD Cranial System)	The 3D photograph was taken of the infant while sitting in a baby 's chair using the 3dMD cranial system. The 3dMD imaging system captures a full 360-degree picture of the head in 1.5 ms.	(Absolute) instrument accuracy not reported. High, proven accuracy of 3dMD cranial system scanner is assumed in this study. Study is focused on reliability: The inter- and intra-rater reliability of 3D photogrammetry proved to be excellent. Given its accuracy, the photographic method can replace anthropometric callipers measurements
Schloesser, R.L., 2011	Body surface area	3D Scanning: stationary desktop setting, structured light, photonic. One scan covers 180 degrees by use of two mirrors (3D-Shape custom-built)	The scanning instrument was a prototype and constructed especially for our study by 3D-Shape, Erlangen, Germany. The measuring system consisted of a projector, two cameras, mirrors and a computer, and used the fringe projection technique with visible light. The infants were examined in a supine position; the hidden parts of the bodies were corrected for using a mathematical factor developed with a baby doll model. The pixels are computed by the software program SLIM3D. Calculation of the surface area is computed with a software program based on Microsoft Excel. The time required for each scan was 0.8 s.	(Absolute) instrument accuracy not reported. High, sufficient accuracy of 3D-Shape scanner is assumed in this study.
Sokolover, N., 2014	Body length	Stereoscopic Vision, stationary, non-photonic, passive still photography (self-built)	An algorithm for stereoscopic measurement of 3D objects from pairs of two-dimensional pictures. The system includes two standard digital still cameras standing on tripods, connected to and controlled by a portable computer that uses the algorithm for 3D measurement.<br>Picture acquisition takes a split second; infants are measured supine and undressed. There is no need to hold the infants in a certain position.<br>After the infant's pictures were taken, six anatomical points of interest were marked on the computer screen, the operator marks on both pictures (in any order) the points of interest to be reconstructed and analysed by the software.	Laboratory tests of stereoscopic vision technique: submillimetre accuracy. The mean of the differences between traditional (length board) and stereoscopic measurements was 0.2 (SD=2.5) mm. The mean of the absolute value of the differences was 2.0 (SD=1.4) mm. With the exception of a single outlier of 7.4 mm (not excluded from data), all differences were less than 5 mm.
Tenhagen, M., 2016	Head: Head circumference, sagittal length, coronal width, cranial volume	3D scanning: handheld structured light 3D scanner, photonic, 360 scan with mosaic move-around-object capture (M4D Scan)	Structured light handheld scanner, the M4D Scan 3D scanner provided by Rodin4D.<br>VXelements (Creaform, Levis, Quebec, Canada) software was used in conjunction with the 3Dcamera during the acquisition to assess the result in real time.<br>Post-processing of 3D-images (offline): First, MeshMixer (Autodesk Research, Toronto, Canada) was used to clean up noise due to surrounding structures. If multiple scans were taken of the same patient at the same time point, the incomplete scans were loaded into 3-matic (Materialise, Leuven, Belgium) where they were registered with each other using a combination of N-point (5–6 manually selected reference anatomical landmarks) and global registration algorithms, before merging them into one complete 3D scan.<br>Second, the seams of the stocking were smoothed out and the holes around the ears were filled to ensure that the volume of the 3D mesh could be calculated properly.<br>Third, the lower border of the patient head model was defined to ensure an easily and consistently identifiable cutting plane.<br>Shape analysis: Statistical shape modelling (SSM) was carried out using the registered 3D surface meshes to compute 3D anatomical mean shapes for the population. All shape analysis was carried out within the Deformetrica shape modelling framework (www.deformetrica.org)<br>For the computed mean shapes from the SSM and for each patient, surface distances between the postoperative and the preoperative models, as well as between the follow-up and the preoperative models, were calculated using VMTK24 (www.vmtk.org).<br>Respective distance colour maps were generated in ParaView25 to visualize and analyse postsurgical local-shape changes.	High (instrument-)accuracy was presumed to be sufficient based on other studies (manufacturer website: accuracy: up to 0,5 mm, standoff distance approx. 40 cm.)<br>Accuracy comparison: Only the cephalic index was compared with CT scan values.<br>Interesting is the method of analysis of head shape: instead of commonly used pointed-based (landmarks), they align the 3D image by a reference plane and measure distances/changes between surfaces of 3D images (e.g., different moments in time) that can be visualised in 'colour maps'.

Tu, L.Y., 2020	Head: Intracranial volume, head volume	3D Scanning: stereophotogrammetric, stationary, 360 degrees scan in one capture. (3dMD Head System)	CT images and 3D photography, polynomial regression modelling. Three-dimensional photographs were acquired using the 3dMDhead System manufactured by 3dMD LLC (Atlanta, Ga.)	Reported accuracy of the prediction model: The authors obtained an average error of 4.07 ± 3.01 (percent ± SD) in estimating the intracranial volume of the patients from three-dimensional photography using the regression model.
Vermeulen, M.J., 2021	Head: Cranial volume	3D Scanning, laser shape digitizer, stationary (desktop), photonic (laser light), 360 degrees scan in one capture (STARscanner)	3-D laser shape digitizer (STARscannerTM, Orthomerica Products Inc, Orlando, FL, USA). After placing the infant in the scanner, a 3-D head shape was captured within a few seconds, by four Class 1 eye-safe lasers and eight cameras. Using specialized software (YETITM Shape Builder and Head Comparison Utility, Vorum Research Corporation, Vancouver, Canada) cranial volume and head circumference were calculated based on predefined anatomical reference planes	not reported. Refers to previous studies that evaluated accuracy and reliability of STARscanner.<br>Author's comments: Method was validated in Burkhardt 2019 study
Wang, J.C., 2000	Body length	2D Linear metric, measure from existing photographs (no device used)	Calculate height of children by photos on which children are standing against and reference object from which size in known. Photographs taken by own camera at home; not described which camera. The size of the reference object is measure on the photo in mm. By the relative metric ratio to a reference object the body height is calculated.	In this study accuracy of 1,5 cm was achieved. The mean differences between the measured and estimated heights were 1.4 cm and 1.5 cm respectively, and both were not statistically significant
Weinberg, S.M., 2006	Head: Face dimensions	3D Scanning:<br>1) photogrammetry, stationary, photonic, structured light, capture from one viewing point (Genex)<br>2) photonic unstructured light, stationary, 180 degrees (ear-to-ear) capture (3dMD Face System)	1. Genex FaceCam 250 imaging system (Kensington, MD), the Genex system projects a structured light pattern onto an object's surface to generate and extract 3D information<br>2. 3dMD MU-4 imaging system (Atlanta, GA), 3dMD system projects a random (i.e., unstructured) light pattern, can accommodate additional cameras (for additional facial views) without the need for supplementary captures and with no reduction in capture speed.<br>Genex and 3dMD systems are both fully automated digital 3D photogrammetric devices capable of very fast (<1 second), high-resolution colour surface captures.	Genex and 3dMD system are sufficiently concordant (relative to one another), accurate (relative to direct anthropometry), and precise to meet the needs of most clinical and basic research designs: mean difference scores (<1 mm) and the generally weak effect size statistics (Range -0.28 to +0.22).
