# Supplementary material for: Body size measuring techniques enabling stress-free growth monitoring of extreme preterm infants inside incubators: A systematic review
Source: PLoS One. 2022 Apr 22;17(4):e0267285. doi: 10.1371/journal.pone.0267285 (PMC9033282; doi:10.1371/journal.pone.0267285)
Supplement: S4 Data — (PDF) [file pone.0267285.s008.pdf]

S4 Data-table. Disturbance of patients during measurement

First Author; year	Type of body size measurement: Body Length (BL), Head Circumference (HC), Head Volume (HV) or Cranial Volume (CrV), etc.	Technology type (device model)	Time needed for measurement, if reported.	Disturbance of patient during measurement: due to preparation, positioning or repositioning	Disturbance of patient during measurement: exposure to light
Andrews, E.T., 2019	Head: Head circumference; Body length	3D Scanning: stereoscopic, photonic, handheld, point-and-shoot capture from one viewing point (SCANIFY)	Not reported	No disturbance e.g., repositioning or preparation was reported. Length measurements were taken from directly above the infant. HC measurements were taken in line with the top of the head (or as close as possible) to obtain an image of the largest possible HC. Author's comments: A light shield mask was placed on top of the infant’s eyes, but they were not repositioned or directly handled for the scan. A "target" was placed next to or on the infant, this caused minimal disruption as the target was made only of laminated paper. The images are taken using the device by dropping the side door of the incubator and capturing the image of the baby without moving or touching them very much. Whilst infants were not repositioned for the image capture sometimes it was necessary to move some items in the incubator or cot for example "octopus toys" or flatten sheets.	Author's comments: The light had several flashes and did disturb some of the infants despite the eye protection and others did not appear to be.
Barbero-García, I., 2017	Head: Cranial shape and size (perimeter)	3D Scanning: 3D photogrammetry, handheld (Smartphone), non-photonic: passive, slow motion video capture, 360 degrees scan by move-around-object capture (Samsung S7 Smartphone)	Between 3 and 5 min. for the preparation and video recording. No time reported for 3D image processing.	Cap with markers (stickers) placed on head. During the video session, the infant was held by an adult. Child held upright or lying providing that all areas of head are visible. Move child to new position during video capture to assure whole head is covered.	Author's comments: No
Barbero-García, I., 2020	Head: Head shape	3D Scanning: 3D photogrammetry, handheld (Smartphone), non-photonic: passive, capture, 360 degrees scan by move-around-object capture	the acquisition time was 1.1 ± 0.17 min. For various users, the acquisition time was higher: 1.5 ± 0.6 min. Postprocessing: The calculation time was 2.5 ± 0.6 min for the expert user and 2.7 ± 0.9 min for different users, many of them with no previous knowledge of the tool.	In vitro study, no patients. However, coded cap with markers must be placed on head. Together with the cap, three stickers are provided. The stickers are similar to the markers present on the cap. The medical staff carrying out the image acquisition are asked to place one of them between the eyes and the others on the left-hand side and right-hand side pre-auricular points.	Author's comments: It is not reported in this paper but in previous ones [10.1016/j.measurement.2018.08.059]. No disturbance and no extra lights are required. Visible light.

Brons, S., 2019	Head: Face dimensions	3D Scanning: stereophotogrammetric, stationary, 360 degrees scan in one capture. (3dMD Cranial System)	The image capture duration was 1.5 milliseconds. On each occasion, approximately four images were obtained within 10 minutes, depending on the subject's cooperation.	Not reported	Not reported. YES? 3dMD uses additional visible light for object illumination
Burkhardt, W., 2019	Head: Total Brain Volume (TBV), Cranial volume (CrV) and head circumference (HC)	3D scanning:<br>1) laser shape digitizer, stationary (desktop), photonic (laser light), 360 degrees scan in one capture (STARscanner)<br>2) structured light projection, stationary, photonic, point-and-shoot capture from one viewing angle (GOM ATOS Triple Scan II)<br>3) structure from motion, handheld, passive image capture, 360 capture by multiple images (Agisoft PhotoScan software)<br>4) laser light sheet scanner, robot arm, photonic (MicroScan 3D – RSI)	1) STARscanner: scan procedure within 3 seconds<br>2) Structure light projection system: not reported<br>3) Structure from motion: not reported	1) STARscanner: the neonate had to be positioned inside the STARscanner: "Infants are placed in the non-invasive laser shape digitizer which captures a 3D infant head shape within 3 s using four Class-I eye-safe lasers"; no specific information is given concerning the use of landmark points or other preparation like caps.<br>2) Structure light projection system: unknown, only used with puppet head<br>3) Structure from motion: unknown, only used with puppet head	YES. STARscanner: four Class-I eye-safe lasers are used
Conkle, J., 2019	Head: Head circumference;<br>Body length,<br>Arm circumference	3D Scanning: structured light (infrared) 3D scanner, photonic, handheld, move-around-object (mosaic) capture (Occipital Structure Sensor with AutoAnthro software)	68 sec for 3D scanner compared to 135 s for manual measurement.	Children over 2 years standing up arms in three positions; children under 2 years lying down in their arms extended away from the torso;<br>Crying: 20% crying during manual measurement and all infants under 2 years. One child (4%) during 3D-scan.	Not reported. From other online source (manufacturer website): infrared
de Jong, G., 2020	Head: Head shape	3D Scanning: stereophotogrammetric, stationary, 360 degrees scan in one capture. (3dMD Cranial System)	Not reported	Not reported	Not reported. YES? 3dMD uses additional visible light for object illumination

Firmansyah, R., 2019	Head: Head circumference	Ultrasonic, distance sensor (self-built)	Not reported	Technology is aimed to be integrated in incubator; no specific preparation needed	NO<br>Author's comments: NO
Geil, M.D., 2008	Head: Head circumference, sellions landmarks at level 3, and cranial vault asymmetry index (CVAI)	3D Scanning, laser shape digitizer, stationary (desktop), photonic (laser light), 360 degrees scan in one capture (STARscanner)	Not reported	Not tested, mannequin foam model, cotton stockinette used leaving face and ears exposed	not tested, mannequin. Introduction mentioned four eye-safe lasers the create circumferential light beams.
Goto, L., 2019	Head: Head and face dimensions	3D Scanning, photogrammetry, stationary (3dMD Face System)	Not reported	Before photographing, each participant was provided with a nylon wig cap to capture the shape of the head and to avoid noise or holes in the 3D data caused by hair. The child was positioned on a highchair that was mounted on a plateau on wheels in order to be able to rotate the child in the respective angles	Not reported. Author's comments: YES, the 3dMD Face system comes with 3 Flash modules.
Ifflaender, S., 2013	Head: Head circumference and Head volume (CrV)	3D Scanning: laser shape digitizer, stationary (desktop), photonic (laser light), 360 degrees scan in one capture (STARscanner)	For scanning the infant has to be placed in the scanner<br>for about 20 seconds. The scanning process lasts about 3 seconds, a period where the infant should not move.<br>Time needed for 3D postprocessing was not reported.	Before the infant is scanned, the infant's head is covered by<br>stockinet to compress and mask the hair while allowing exposure<br>of the face and both ears. Otherwise, no other preparation is<br>required.	YES, eye-safe laser light
Linz, C., 2014	Head: Head volume (Cranial volume), head circumference, width, length, max width and length (Cranial index), total cranial volumes of Q1 - Q4	3D Scanning: stereophotogrammetric, stationary, 360 degrees scan in one capture. (3dMD Cranial System)	Not reported.<br>3D postprocessing is needed to acquire the measurements, postprocessing time not reported.	To avoid artifacts due to hair, each<br>infant was fitted with a tight nylon cap before recording	Not reported. YES? Ambient light bars? (manufacturer's website)
Martini, M., 2018	Head: Head circumference; ear-to-ear over the head distance; maximal cranial length measurement; cranial volume	3D Scanning: structured light, photonic, stationary*? (3D-Shape*)<br>* device model not reported	Not reported	Patient is positioned in the FaceSCAN3D scanner	Not reported. Based on FaceSCAN3D manufacturer website: YES, structured light, extra flashlight

Meyer-Marcotty, P., 2014	Head: CrV	3D Scanning: stereophotogrammetric, stationary, 360 degrees scan in one capture. (3dMD Cranial System)	Recording time of 1.5ms. Photo-optical scanner; No radiation was used.<br>Post processing of the 3D data "To align the 3D datasets in virtual space" was needed; time needed for this postprocessing was not reported.	Each infant wore a nylon cap to prevent artefacts due to their scalp hair.	Not reported. Extra visible light (flash)?
Meyer-Marcotty, P., 2018	Head: CrV	3D Scanning: stereophotogrammetric, stationary, 360 degrees scan in one capture. (3dMD Cranial System)	Recording time of <1.5 ms	Each infant wore a nylon cap to prevent artefacts due to their scalp hair.	Not reported. Extra visible light (flash)?
Nahles, S., 2018	Head: Head circumference, Head length, Head width, head diagonals	3D Scanning: handheld, structured light, photonic, can make 360 degrees scan with mosaic move-around-object capture (OMEGA)	The measurement process with the scan method required a mean of 579.6 s (SD 202.8 s) with a minimum of 323 s and a maximum time of 1034 s, whereas with the manual anthropometric method, a mean time of 180.5 s (SD 202.8 s; min 95 s; max 330 s) was documented.<br>Time needed for 3D scan much larger compared to traditional methods. Time for scan was recorded until acquisition of 3D image was finished. It is not reported how and how much time needed to derive the metric parameters from the 3D image.	Black Cape; reflector dots with a diameter of 6 mm; nylon hood; At the beginning six reflector dots were fixed at definitive points of the face or the hood as important point for the creation of the virtual 3D-model. The positions of the reflector dots are the same as those used in the manual for conventional assessment. Thereafter, the white nylon hood was placed on the head, leaving the face and ears uncovered. The black cap was then folded to prevent interference effects;	YES, pattern of visible light
Ritschl, L.M., 2018	Head: Face dimensions, perinasal area	3D Scanning: photogrammetry, photonic, handheld, point-and-shoot capture gives 3D scan from one viewing angle. (SCANIFY)	SCANIFY capture speed 0.1 s	Not reported	xenon flashlights; LED searchlights
Santander, P., 2019	Head: HC, Head shape, CrV	3D Scanning: Stereophotogrammetry with added flash, handheld, point-and-shoot. 10 separate captures needed to assemble a 360 degrees scan (VECTRA H1)	Each capture took 1.5 ms and according to the manufacturer, recharging the flashlight needed 5 s. According to our study protocol, ten images per head were taken, resulting in a mean duration of 3:34 ± 2:05 min per infant.	"The preterm infants were equipped with eye protectors and a nylon cap. Captures were acquired on the neonatal ward with the infant lying in their bed or incubator or during kangarooing by either walking around the infant or during different phases of patient care. No positioning of the infant solely for study purposes was needed."	YES, flashlight

Schaaf, H., 2010	Head: Cranial shape/volume/size, cranial vault asymmetry index (CVAI)	3D Scanning: photogrammetry, stationary, 360 degrees scan in one capture. (3dMD Cranial System)	Duration of capture 1.5 ms	Picture showed cap and ponts attached to child. Positioning: child is sitting on parent’s lap facing the physical assistant.	Not reported. YES? 3dMD uses additional visible light for object illumination
Schloesser, R.L., 2011	Body surface area	3D Scanning: stationary desktop setting, structured light, photonic. One scan covers 180 degrees by use of two mirrors (3D-Shape custom-built)	Not reported	Patient has to be positioned on the instrument, fully naked. The hair was covered with a light skull cap.	YES, Visual light fringes are projected on the patient, for 0.8s.
Sokolover, N., 2014	Body length	Stereoscopic Vision, stationary, non-photonic, passive still photography (self-built)	Author's comments: Total measurement procedure time was few minutes. measurement by itself- seconds.	Possible repositioning of patient. Patient should be naked for best view on body points Author's comments: yes. Babies were undressed, supine. No need for other repositioning. but handling of babies was needed. Could be done in own bed but was not tried through incubator walls (although theoretically and laboratory tested possible).	Author's comments: no additional lighting was used. Spec- passive light acquisition (still photography).
Tenhagen, M., 2016	Head: Head circumference, sagittal length, coronal width, cranial volume	3D scanning: handheld structured light 3D scanner, photonic, 360 scan with mosaic move-around-object capture (M4D Scan)	Time needed for a scan 5-10 min. Postprocessing: manual process requiring between 45 and 60 operator minutes per 3D scan.	A white nylon stocking (Beagle Orthopaedic, Blackburn, UK) was placed on the head of the patients to overcome the difficulties of the scanner in capturing hair.	YES, pattern of visible light (white LED light)
Tu, L.Y., 2020	Head: Intracranial volume, head volume	3D Scanning: stereophotogrammetric, stationary, 360 degrees scan in one capture. (3dMD Head System)	3D capture in approximately 1.5 msec	Author's comments: For these studies, data were obtained with the 3dMD Head System, which requires the patient to be positioned on a chair in the centre of calibrated cameras. Other portable devices can be used to capture the data.	Not reported. YES? 3dMD uses additional visible light for object illumination. Author's comments: Our software is not specific to 3DMD data.
Vermeulen, M.J., 2021	Head: Cranial volume	3D Scanning, laser shape digitizer, stationary (desktop), photonic (laser light), 360 degrees scan in one capture (STARscanner)	3D capture "within a few seconds"	Not reported	YES, four Class-I eye-safe lasers

Wang, J.C., 2000	Body length	2D Linear metric, measure from existing photographs (no device used)	Not reported	NO	NO
Weinberg, S.M., 2006	Head: Face dimensions	3D Scanning:<br>1) photogrammetry, stationary, photonic, structured light, capture from one viewing point (Genex)<br>2) photonic unstructured light, stationary, 180 degrees (ear-to-ear) capture (3dMD Face System)	The 3dMD system capture time 400 ms and Genex capture time <2 sec.; two separate captures needed (frontal and 45 degree left)	Not reported	YES, pattern of visible light
