# Supplementary material for: Body size measuring techniques enabling stress-free growth monitoring of extreme preterm infants inside incubators: A systematic review
Source: PLoS One. 2022 Apr 22;17(4):e0267285. doi: 10.1371/journal.pone.0267285 (PMC9033282; doi:10.1371/journal.pone.0267285)
Supplement: S5 Data — (PDF) [file pone.0267285.s009.pdf]

S5 Data-table. Assessment of the techniques' suitability for measuring the body size of preterm infants lying in an incubator

First Author; Year	Type of body size measurement:<br>Body Length (BL), Head Circumference (HC), Head Volume (HV) or Cranial Volume (CrV), etc.	Technology (device type)	Infants measured in incubator?<br>0= not reported or not feasible<br>1= not reported but reasonable belief in feasibility<br>2= reported measurements of infants in incubator<br>3= reported measurements of ventilated infants in incubator	Accuracy clinically sufficient?<br>0= no data available in study or reported as not sufficient<br>1= as 0, but reasonable belief in feasibility<br>2= reported or assessed as sufficient<br>3= sufficient and not influenced by user-actions	Measures through incubator cover?<br>0= not reported or not feasible<br>1= not reported but reasonable belief in feasibility through open doors or closed cover<br>2= yes, through open doors<br>3= yes, through closed cover	Disturbance by preparation or repositioning?<br>0= yes or not reported<br>1= yes, but reasonable belief that extra handling could be combined with routine care<br>2= yes, but extra handling was combined with routine care<br>3= no preparation or reposition needed	Measures relevant body size parameters?<br>0= no reported measurements of BL, HC, or CrV<br>1= BL and/or HC<br>2= CrV<br>3= CrV, HC, and BL with one device	Resulting Suitability Class:<br>0= not suitable<br>1= potentially suitable<br>2= suitable<br>3= ideal device	Suitability Class, with reviewers' motivation and remarks
Andrews, E.T., 2019	Head: Head circumference; Body length	3D Scanning: stereoscopic, photonic, handheld, point-and-shoot capture from one viewing point (SCANIFY)	3	1	2	2	1	1	Potentially suitable.<br>Could be suitable, if accuracy could be made sufficient: "discrepancy between image captured measured length and manually measured length....,Should be further studied". Also, some difficulties to measure ventilated or respiratory supported infants.<br>Handheld point-and-shoot 3D-camera, captures 3D image from one viewing point. Not sure if camera can capture through transparent incubator cover without problems. Contact with author: "The images are taken using the device by dropping the side door of the incubator and capturing the image of the baby without moving or touching them very much".<br>Reported technical problems (23 scans were not able to be measured due to technical insufficiency of the images obtained 21 HC and 2 length).<br>3D data from one viewing point can give blind spots and lacks 3D data from parts not visible from camera viewing point, which can make it difficult to derive body length and HC.<br>Possible problems by measuring ventilated infants using hat for fixation. "Eighteen of the 21 technically insufficient HC scans were performed on infants receiving continuous positive airway pressure (CPAP) respiratory support who wore a uniformly white CPAP-securing hat which decreased image capture quality."<br>Author's comments: The uniformally white hat required to attach

									CPAP made it difficult for the camera device to accurately represent the infant in 3D.
Barbero-García, I., 2017	Head: Cranial shape and size (perimeter)	3D Scanning: 3D photogrammetry, handheld (Smartphone), non-photonic: passive, slow motion video capture, 360 degrees scan by move-around-object capture (Samsung S7 Smartphone)	1	2	1	1	2	1	Potentially suitable.<br>Image capturing by smartphone via slow-motion video could be promising for patients inside incubators because a smartphone is compact and can be easily moved around an object. Big limitation now are the reference marks (sticker with short metric scale) placed on the head, essential to create an accurate 3D-mesh.Time needed for image capturing is relatively short (3-5 minutes in this study). But filming from outside the incubator's cover may give complications because of the visual distortions of the transparent cover. But because of the compactness of the smartphone, capturing could be done from inside the incubator? But that would compromise hygienics. Slow motion video capturing could allow for some movement of the objected that is captured, but lighting conditions must be good. The process of creating a 3D-mesh from the slow-motion video is very elaborated. Time needed from slow motion video acquisition to completed 3D-mesh was not reported. It involves multiple steps, and camera lens calibration has to be taken into account. They claim accuracy better than 1mm (based on three distances measured). Comparison of distances measured by calliper and 3D model differ in the range of 1-7 mm (table 4) on diagonals, and up to 30 mm on circumference (perimeter).<br>Author's comments: The study was a proof of concept, the processing time was high at that time, around 1 hour. However, the methodology (creating 3D mesh from video) is fully automatic now. (article: Fully automatic smartphone-based photogrammetric 3D modelling of infant’s heads for cranial deformation analysis)
Barbero-García, I., 2020	Head: Head shape	3D Scanning: 3D photogrammetry, handheld (Smartphone), non-photonic: passive, capture, 360 degrees scan by move-around-object capture	1	2	1	1	2	1	Potentially suitable. Follow-up of the 2017 study of same author. Image capturing by smartphone could be promising for patients inside incubators because a smartphone is compact and can be easily moved around an object. Time needed for image capturing is relatively short (3-5 minutes in this study). The described technology allow for some movement of the objected that is captured.<br>Limitations:<br>a)The needed cap with markers is the limiting factor for suitability: Placing the cap cause stress to the preterm infants; Size of caps useful for preterm infants? 131 markers placed on cap for extremely low birthweight infants?<br>b)Low light conditions of incubator. Needed lighting conditions when acquiring images?

									c) The limited space of the incubator: Filming from outside the incubator's cover may give complications because of the visual distortions of the transparent cover. But because of the compactness of the smartphone, capturing could be done from inside the incubator? What is the minimal distance of smartphone to object? Smartphone inside incubator would compromise hygienics. How to capture backside of head?<br>Author's comments: I don't think it would be possible to get the required image coverage with the patient inside an incubator. The cap could be redesigned for smaller infants but right now it is too big.
Brons, S., 2019	Head: Face dimensions	3D Scanning: stereophotogrammetric, stationary, 360 degrees scan in one capture. (3dMD Cranial System)	0	2	0	0	2	0	Not suitable.<br>3dMD cranial system scanner too large for use at incubator, and no information if it could measure through incubator cover
Burkhardt, W., 2019	Head: Total Brain Volume (TBV), Cranial volume (CrV) and Head circumference (HC)	3D scanning:<br>1) laser shape digitizer, stationary (desktop), photonic (laser light), 360 degrees scan in one capture (STARscanner)<br>2) structured light projection, stationary, photonic, point-and-shoot capture from one viewing angle (GOM ATOS Triple Scan II)<br>3) structure from motion, handheld, passive image capture, 360 capture by multiple images (Agisoft PhotoScan software)<br>4) laser light sheet scanner, robot arm, photonic (MicroScan 3D – RSI)	0	2	0	0	2	0	Not suitable.<br>All stationary scanners that were evaluated in this study are not suitable for measuring premature infants at a intensive care, lying inside an incubator. Structure from Motion could work, if used camera is very compact, but no details were reported about used camera.<br>Author's comments: Future approach: use of a multi-camera-system to reduce time to capture images.

Conkle, J., 2019	Head: Head circumference; Body length, Arm circumference	3D Scanning: structured light (infrared) 3D scanner, photonic, handheld, move-around-object (mosaic) capture (Occipital Structure Sensor with AutoAnthro software)	1	1	1	1	1	1	Potentially suitable.<br>Handheld sensor, to be attached to tablet or phone. Potential, if scan can be done through (openings of) incubator cover. Or from inside the cover (iPhone could be compact enough), but required distance to object could then be a problem. Moving infant (long scan time, mosaic capture), incubator cover, required light conditions can give problems. The article refers to a 2018 article in which accuracy is verified, so the article is an validation with the assumption that accuracy is okay.
de Jong, G., 2020	Head: Head shape	3D Scanning: stereophotogrammetric, stationary, 360 degrees scan in one capture. (3dMD Cranial System)	0	2	0	0	0	0	Not applicable.<br>Study into automatic diagnose with artificial intelligence. Used 3D scanner is stationary scanner 3dMD cranial system, not suitable
Firmansyah, R., 2019	Head: Head circumference	Ultrasonic, distance sensor (self-built)	0	0	0	0	0	0	Not suitable.<br>Although the technology is aimed at (premature) infants inside an incubator. Technology readiness level too low. Unclear if and how head circumference is measured. Its seems that 'diameter' is measured rather than circumference. (in fig. 11. 'monitoring diameter' is showed as header in the graph) A ruler is used as reference instrument. The ultra sound distance sensors can measure the width of a intersection of the head that is positioned between the two US sensors. From width of a intersection one could calculate circumference of the section, representing the intersection by a circle, but this is not described. From the five measured data and comparison data three data differ 1 cm, two data zero cm: an inaccuracy of 1 cm.
Geil, M.D., 2008	Head: Head circumference, sellions landmarks at level 3, and cranial vault asymmetry index (CVAI)	3D Scanning, laser shape digitizer, stationary (desktop), photonic (laser light), 360 degrees scan in one capture (STARscanner)	0	2	0	0	2	0	Not suitable.<br>STARscanner is a desktop, stationary scanner, not suitable to scan infants inside incubator. Dummy, foam model used for invitro test, no real patients, unable to draw conclusions for clinical use in NICU setting, little detailed information concerning possible clinical use. STARscanner is used in many other studies, with proven accuracy. Interesting conclusion: 3D measurements might be more valuable than manual.
Goto, L., 2019	Head: Head and face dimensions	3D Scanning, photogrammetry, stationary (3dMD Face System)	0	2	0	0	1	0	Not suitable.<br>3dMD scanner has proven accuracy for anthropometric measurements, but needed distance (130 cm? Fig.2) to object and size of stationary device too large for use at incubator bed-space.

Ifflaender, S., 2013	Head: head circumference and head volume (CrV)	3D Scanning: laser shape digitizer, stationary (desktop), photonic (laser light), 360 degrees scan in one capture (STARscanner)	0	2	0	0	2	0	Not suitable.<br>The STARscanner is a 'desktop', stationary scanner, unsuitable for measurements of patients inside incubators. Premature infants were scanned, but outside incubator.
Linz, C., 2014	Head: Head volume (Cranial volume), Head circumference, width, length, max width and length (Cranial index), Total cranial volumes of Q1 - Q4	3D Scanning: stereophotogrammetric, stationary, 360 degrees scan in one capture. (3dMD Cranial System)	0	2	0	0	2	0	Not suitable.<br>Stationary scanner is too large for use in or around incubator. The used 3D scanner and 3D analysing software from 3dMD are state of the art: accurate, fast.
Martini, M., 2018	Head: Head circumference; Ear-to-ear over the head distance; Maximal cranial length measurement; Cranial volume	3D Scanning: structured light, photonic, stationary*? (3D-Shape*)<br>* device model not reported	0	2	0	0	2	0	Not suitable.<br>It is assumed that the optical 3D sensor used is the 3D-Shape FaceSCAN3D. This device is too large to be used inside or around incubators.
Meyer-Marcotty, P., 2014	Head: CrV	3D Scanning: stereophotogrammetric, stationary, 360 degrees scan in one capture. (3dMD Cranial System)	0	2	0	0	2	0	Not suitable.<br>The scanner of 3dMD is accurate and fast. It is a stationary, floor positioned scan unit, the whole size (Figure 1a.) is too large to use around an incubator bed-space.
Meyer-Marcotty, P., 2018	Head: CrV	3D Scanning: stereophotogrammetric, stationary, 360 degrees scan in one capture. (3dMD Cranial System)	0	2	0	0	0	0	Not suitable.<br>The scanner of 3dMD is accurate and fast. It is a stationary, floor positioned scan unit, the whole size is too big to use around an incubator bed-space. Identical to Meyer-Marcotty 2014.
Nahles, S., 2018	Head: Head circumference, Head length, Head width, Head diagonals	3D Scanning: handheld, structured light, photonic, can make 360 degrees scan with mosaic move-around-object capture (OMEGA)	1	2	1	1	1	1	Potentially suitable<br>Providing 3D-image acquisition is possible through (openings of) transparent cover of incubator. Handheld scanner (OMEGA) is too big (fig. 4) to use inside incubator. Also: Distance 30-40 cm needed between scanner and subject. Mosaic acquisition difficult with moving objects?

Ritschl, L.M., 2018	Head: Face dimensions, Perinasal area	3D Scanning: photogrammetry, photonic, handheld, point-and-shoot capture gives 3D scan from one viewing angle. (SCANIFY)	0	2	0	0	0	0	Not suitable, as used in this study.<br>However, the SCANIFY device, is also used in Andrews 2019. In this study only 3D capture of face. SCANIFY scanner: Limited viewing angle, suitable for the purpose of capture of face. Accuracy seems acceptable for body size purpose (<1mm). But limited if 360 degrees scan are required.<br>As concluded from the Andrews 2019 study: SCANIFY scanner could be used for patients inside incubators, but ventilated patients are difficult to measure, according to Andrews 2019. But not sure if possible through transparent cover (flashlight and LED guiding lights).
Santander, P., 2019	Head: HC, Head shape, CrV	3D Scanning: Stereophotogrammetry with added flash, handheld, point-and-shoot. 10 separate captures needed to assemble a 360 degrees scan (VECTRA H1)	3	2	2	2	2	2	Suitable.<br>The study measures preterm infants in incubators: "...some of the 3D image captures was done with the infant lying in their incubator"<br>But ventilated or respiratory supported infants the ventilation/ gastric tubes can disturb 3D image. Capture is NOT possible through transparent cover of incubator.<br>Disturbance: "The preterm infants were equipped with eye protectors and a nylon cap. No positioning of the infant solely for study purposes was needed."<br>Accuracy: The accuracy and precision is very high. For a full 3D volume scan 10 image captures from 10 different viewing angles are necessary. For a 360 degrees scan it would be necessary to reposition the infant if lying in the incubator.<br>Author's comments: Imaging is possible through the openings of the incubator. Due to the reflection of the flashlight, the image through the transparent plastic is not possible
Schaaf, H., 2010	Head: Cranial shape/volume/size, Cranial vault asymmetry index (CVAI)	3D Scanning: photogrammetry, stationary, 360 degrees scan in one capture. (3dMD Cranial System)	0	1	0	0	2	0	Not suitable.<br>The 3dMD system has proven accuracy and fast capture time. But not suitable for preterm infants inside incubators, the stationary nature, size of instrument, hinders it usefulness in or around incubator.
Schloesser, R.L., 2011	Body surface area	3D Scanning: stationary desktop setting, structured light, photonic. One scan covers 180 degrees by use of two mirrors (3D-Shape custom-built)	0	2	0	0	0	0	Not suitable.<br>The technology provide almost full body 3D scans, by use of two mirrors to capture the two sides of the body. This instrument set-up cannot be used to measure infants inside incubators. Interesting is the method where the hidden parts (in the shadow of the 3D cameras) are corrected: "the hidden parts of the bodies were corrected for using a mathematical factor developed with a baby doll model". In this way they could compute/estimate the BSA without having a full 360 degrees body scan.

Sokolover, N., 2014	Body length	Stereoscopic Vision, stationary, non-photonic, passive still photography (self-built)	1	2	3	2	1	2	Suitable.<br>Although the study did not measure infants inside incubators, lab-test proofs feasibility for measuring through the closed cover. Infant must be naked and correctly positioned for cameras to reveal good line of sight on relevant body points: this can be done during routine diaper change.<br>"It can potentially be used to measure new-borns inside incubators. This will enable length measurements of little preemies and sick neonates who are often not measured." This could include ventilated or respiratory supported infants.<br>Contact with author: "We specifically developed a system intended to measure from (from outside) babies inside the incubator, and it works. We calculated the distortion due to refraction by the plastic, and it turned out to be negligible."<br>Reported accuracy and precision is sufficient to replace manual measuring instruments. Accuracy is user-dependent because of hand selection of body points on the two separate images.
Tenhagen, M., 2016	Head: Head circumference, Sagittal length, Coronal width, Cranial volume	3D scanning: handheld structured light 3D scanner, photonic, 360 scan with mosaic move-around-object capture (M4D Scan)	1	2	1	1	2	1	Potentially suitable.<br>The M4D Handheld scanner might be suitable, if image acquisition is possible through (openings of) transparent cover of incubator. The M4D Scan handheld scanner is very big to use inside an incubator (And the scanner has to be moved around the object with a stand-off distance of approx. 40 cm). Not known if it could measure through the transparent cover.<br>Author's comments: 3D Structured scanners should be able to scan through transparent objects (e.g. incubator). The reflection of light caused by the shiny surface of the incubator might cause artifacts in the 3D scan. The biggest issue while making a 3D scan with the infant in the incubator is maintaining the effective scanning range of the handheld scanner. The stand off distance is quite forgiving, and not exactly the mentioned 40cm.
Tu, L.Y., 2020	Head: Intracranial volume, Head volume	3D Scanning: stereophotogrammetric, stationary, 360 degrees scan in one capture. (3dMD Head System)	0	2	0	0	2	0	Not applicable.<br>The study does not evaluate a measuring technique, but a calculation method for predicting intra-cranial volume from 3D photography data of cranial volume.<br>Author's comments: It could be used with ventilated patients using a hand-held data capturing device
Vermeulen, M.J., 2021	Head: Cranial volume	3D Scanning, laser shape digitizer, stationary (desktop), photonic (laser light), 360 degrees scan in one	0	2	0	0	2	0	Not applicable.<br>Aim of studie is to develop CrV growth reference charts, and uses CrV data from STARscanner, from the Burkhardt 2019 and Ifflaender 2013 studies (Dresden, Germany).

		capture (STARscanner)							
Wang, J.C., 2000	Body length	2D Linear metric, measure from existing photographs (no device used)	1	1	1	1	1	1	Potentially suitable.<br>The technique as used in this study is not suitable, however the method is similar to 2D vision technology, metric estimation of an object by known size of a reference object on the same 2D image, which can be very accurate Further research is needed to explore if such a 2D metric techniques is feasible to measure body length of infants in an incubator. The technique as deployed in this study is suitable for 2D objects, where object and reference object are in the same plane, same distance to the camera.
Weinberg, S.M., 2006	Head: Face dimensions	3D Scanning:<br>1) photogrammetry, stationary, photonic, structured light, capture from one viewing point (Genex)<br>2) photonic unstructured light, stationary, 180 degrees (ear-to-ear) capture (3dMD Face System)	0	1	0	0	2	0	Not suitable.<br>Both scanners are stationary scanners, and are likely too large to use in or around incubator. (External found pictures/specs of Genex FaceCam 250 imaging system: device is stationary, too big). Both systems showed sufficient precision and accuracy, but only mannequin heads were measured, no patients.
