# Supplementary material for: Body size measuring techniques enabling stress-free growth monitoring of extreme preterm infants inside incubators: A systematic review
Source: PLoS One. 2022 Apr 22;17(4):e0267285. doi: 10.1371/journal.pone.0267285 (PMC9033282; doi:10.1371/journal.pone.0267285)
Supplement: S6 Data — (PDF) [file pone.0267285.s010.pdf]

S5 Data-table. Techniques assessed as Suitable or Potentially Suitable for measuring body size of preterm infants lying in incubators

First Author; Year	Technology (device type)	Infants measured in incubator?<br>0= not reported or not feasible<br>1= not reported but reasonable belief in feasibility<br>2= reported measurements of infants in incubator<br>3= reported measurements of ventilated infants in incubator	Accuracy clinically sufficient?<br>0= no data available in study or reported as not sufficient<br>1= as 0, but reasonable belief in feasibility<br>2= reported or assessed as sufficient<br>3= sufficient and not influenced by user-actions	Measurements through incubator cover?<br>0= not reported or not feasible<br>1= not reported but reasonable belief in feasibility through open doors or closed cover<br>2= yes, through open doors<br>3= yes, through closed cover	Disturbance by preparation or repositioning?<br>0= yes or not reported<br>1= yes, but reasonable belief that extra handling could be combined with routine care<br>2= yes, but extra handling was combined with routine care<br>3= no preparation or reposition needed	Can measure relevant body size parameters?<br>0= no reported measurements of BL, HC, or CrV<br>1= BL and/or HC<br>2= CrV<br>3= CrV, HC, and BL with one device	Suitability Class	Suitability Class, additional remarks<br>0= not suitable<br>1= potentially suitable<br>2= suitable<br>3= ideal device
Santander, P., 2019	3D Scanning: Stereophotogrammetry with added flash, handheld, point-and-shoot. 10 separate captures needed to assemble a 360 degrees scan (VECTRA H1)	3<br>"...some of the 3D image captures was done with the infant lying in their incubator"<br>But ventilated or respiratory supported infants the ventilation/ gastric tubes can disturb 3D image.	2<br>Accuracy: The accuracy and precision is very high.	2<br>Capture is NOT possible through transparent cover of incubator.<br>Author's comments: Imaging is possible through the openings of the incubator. Due to the reflection of the flashlight, the image through the transparent plastic is not possible	2<br>Disturbance: "The preterm infants were equipped with eye protectors and a nylon cap. No positioning of the infant solely for study purposes was needed." For a 360 degrees scan it would be necessary to reposition the infant if lying in the incubator.	2<br>HC and CrV with one device.<br>“For a full 3D volume scan 10 image captures from 10 different viewing angles are necessary”.	2	Suitable.<br>However, the image capture method seems unpractical for use at a NICU. Furthermore, 3D postprocessing to derive body size data is complicated and time-consuming
Sokolover, N., 2014	Stereoscopic Vision, stationary, non-photonic, passive still photography (self-built device)	1<br>"It can potentially be used to measure new-borns inside incubators. This will enable length measurements of little preemies and sick neonates who are often not measured." This could include ventilated or respiratory supported infants.	2<br>Reported accuracy and precision is sufficient to replace manual measuring instruments. Accuracy is user-dependent because of hand selection of body points on the two separate images.	3<br>Although the study did not measure infants inside incubators, lab-test proofs feasibility for measuring through the closed cover. Contact with author: "We specifically developed a system intended to measure from (from outside) babies inside the incubator, and it works. We calculated the distortion due to refraction by the plastic, and it turned out to be negligible."	2<br>Infant must be naked and correctly positioned for cameras to reveal good line of sight on relevant body points: this can be done during routine diaper change.	1<br>Only BL	2	Suitable.<br>Measuring through the closed cover is a strong point. Another strong point is the ease of use and instant result of body length, without any postprocessing time.

Andrews, E.T., 2019	3D Scanning: stereoscopic, photonic, handheld, point-and-shoot capture from one viewing point (SCANIFY[a])	3<br><br>Possible problems by measuring ventilated infants using hat for fixation. "Eighteen of the 21 technically insufficient HC scans were performed on infants receiving continuous positive airway pressure (CPAP) respiratory support who wore a uniformly white CPAP-securing hat which decreased image capture quality."<br>Author's comments: The uniformally white hat required to attach CPAP made it difficult for the camera device to accurately represent the infant in 3D.	1<br><br>Reason to score 1: "discrepancy between image captured measured length and manually measured length....,Should be further studied".	2<br><br>Not sure if camera can capture through transparent incubator cover without problems. Contact with author: "The images are taken using the device by dropping the side door of the incubator …".	2<br><br>Contact with author: "The images are taken using the device by … capturing the image of the baby without moving or touching them very much".	1<br><br>BL and HC with one device is a strong point of the technique. However, the point-and-shoot 3D-camera, captures 3D image from one viewing point, resulting in blind spots, lacking 3D data from parts not visible from camera viewing point, which can make it difficult to derive body length and HC.	**1**	Potentially suitable.<br><br>The study scores best on measured ventilated infants in incubators. This technique would score as suitable, if accuracy would have been sufficient.<br>The point-and-shoot capture through open doors, one capture for HC and one for BL, could fit in NICU routine care.<br>Reported technical problems: 23 scans were not able to be measured due to technical insufficiency of the images obtained (21 HC and 2 length). No information about needed postprocessing-time to derive data was reported.
Barbero-García, I., 2017	3D Scanning: 3D photogrammetry, handheld, non-photonic: passive, slow motion video capture, 360 degrees scan by move-around-object capture (Samsung S7 Smartphone)	1<br><br>Image capturing by smartphone via slow-motion video could be feasible because a smartphone is compact and can be easily moved around an object. Time needed for image capturing is relatively short (3-5 minutes in this study). Slow motion video capturing could allow for some movement of the object that is captured, but lighting conditions must be good.	2<br><br>They claim accuracy better than 1mm (based on three distances measured). Comparison of distances measured by calliper and 3D model differ in the range of 1-7 mm (table 4) on diagonals, and up to 30 mm on circumference (perimeter).	1<br><br>Filming from outside the incubator's cover may give complications because of the visual distortions of the transparent cover. Capturing could be done from inside the incubator? But that would compromise hygienics.	1<br><br>Big limitation now are the reference marks (sticker with short metric scale) placed on the head, essential to create an accurate 3D-mesh	2<br><br>HC and CrV with one device. However, the process of creating a 3D-mesh from the slow-motion video is very elaborated and time-consuming. Author's comments: The study was a proof of concept, the processing time was high at that time, around 1 hour. However, the methodology (creating 3D mesh from video) is fully automatic now.	**1**	Potentially suitable.<br><br>This study was followed up by a study (Barbero-García, I., 2020) automating the postprocessing process.

Barbero-García, I., 2020	3D Scanning: 3D photogrammetry, handheld non-photonic: passive, capture, 360 degrees scan by move-around-object video capture (Smartphone)	1<br>Could be promising for patients inside incubators because a smartphone is compact and can be easily moved around an object. Time needed for image capturing is relatively short (3-5 minutes in this study). The described technology allow for some movement of the objected that is captured.	2<br>Claimed accuracy better than 1 mm	1<br>Filming from outside the incubator's cover may give complications because of the visual distortions of the transparent cover. Author's comments: I don't think it would be possible to get the required image coverage with the patient inside an incubator.<br>Capturing could be done from inside the incubator? But that would compromise hygienics.	1<br>The needed cap with markers is the limiting factor for suitability: Placing the cap cause stress to the preterm infants; Author's comments: The cap could be redesigned for smaller infants but right now it is too big.	2<br>HC and CrV with one device	**1**	Potentially suitable.<br>Follow-up of the 2017 study of same author: this study fully automates the method to create a 3D mesh from smartphone video capture.
Conkle, J., 2019	3D Scanning: structured light (infrared) 3D scanner, photonic, handheld, move-around-object capture (Occipital Structure Sensor with AutoAnthro software)	1<br>Handheld sensor, to be attached to tablet or phone could be compact enough for capture in NICU-setting. However, moving infant (long scan time, move-around-object capture), incubator cover, required light conditions can give problems.	1<br>The article refers to a 2018 article in which accuracy is verified, so the article is an validation with the assumption that accuracy is sufficient.	1<br>Further research should proof if move-around-object capture is feasible through open doors or closed incubator cover. Or from inside the cover (iPhone could be compact enough), but required distance to object could then be a problem.	1<br>Infants must be in line-of-sight, should be feasible to combine with routine care handling	1<br>HC and BL with one device. Unknown if 3D volumetric data is feasible.	**1**	Potentially suitable.<br>No information about needed postprocessing to derive body size data.
Nahles, S., 2018	3D Scanning: handheld, structured light, photonic, can make 360 degrees scan with move-around-object capture (OMEGA[b])	1<br>Move-around-object acquisition difficult with moving objects?	2<br>According to the manufacturer, the instrument is accurate to within 0.5 mm over the entire surface.	1<br>Providing 3D-image acquisition is possible through (openings of) transparent cover of incubator. Handheld scanner (OMEGA) is too big (fig. 4) to use inside incubator. Also: Distance 30-40 cm needed between scanner and subject.	1<br>The needed cap with reflector dots is the limiting factor for suitability: Placing the cap cause stress to the preterm infants	1<br>HC only	**1**	Potentially suitable.

Tenhagen, M., 2016	3D scanning: handheld structured light 3D scanner, photonic, 360 scan with move-around-object capture (M4D Scan[b])	1<br><br>The M4D Handheld scanner might be suitable, if image capture is feasible through (openings of) transparent cover. Scanning inside the cover seems not feasible because of the large size of the scanner. Author's comments: …The biggest issue while making a 3D scan with the infant in the incubator is maintaining the effective scanning range of the handheld scanner. The standoff distance is quite forgiving, and not exactly the mentioned 40cm.	2<br><br>High instrument-accuracy was presumed to be sufficient based on other studies (manufacturer website: accuracy: up to 0,5 mm, standoff distance approx. 40 cm.)	1<br><br>Not known if it could measure through the transparent cover. Author's comments: 3D Structured scanners should be able to scan through transparent objects (e.g. incubator). The reflection of light caused by the shiny surface of the incubator might cause artifacts in the 3D scan…	1<br><br>Infants must be in line-of-sight during image capture, should be feasible to combine with routine care handling	2<br><br>HC and CrV with one device	**1**	Potentially suitable.
Wang, J.C., 2000	2D Linear metric, measure from existing photographs (no device used)	1<br><br>The capture of one 2D image to derive BL seems feasible for a NICU setting.	1<br><br>The technique as used in this study is not suitable, however the method is similar to 2D vision technology, metric estimation of an object by known size of a reference object on the same 2D image, which can be very accurate.	1<br><br>2D image capturing should be feasible through transparent cover, preferably without any additional light.	1<br><br>Infants must be in line-of-sight during image capture, should be feasible to combine with routine care handling	1<br><br>BL only	**1**	Potentially suitable.<br>The technique as deployed in this study is suitable for 2D objects, where object and reference object are in the same plane, same distance to the camera. Further research is needed to explore if a 2D linear metric technique is feasible to measure body length of infants in an incubator.

[a]The Scanify scanner was also used in Ritschl, L.M., 2018, but only aimed at face dimensions.

[b]The OMEGA and M4D handheld 3D scanners seem technically identical.
